# An empirical mean-field model of symmetry-breaking in a turbulent wake

**DOI:** 10.1126/sciadv.abm4786

**Published:** 2022-05-11

**Authors:** Jared L. Callaham, Georgios Rigas, Jean-Christophe Loiseau, Steven L. Brunton

**Affiliations:** 1 Department of Mechanical Engineering, University of Washington, Seattle, WA 98195, USA.; 2 Department of Aeronautics, Imperial College London, London SW7 2AZ, UK.; 3 Arts et Métiers Institute of Technology, CNAM, DynFluid, HESAM Université, F-75013 Paris, France.

## Abstract

Improved turbulence modeling remains a major open problem in mathematical physics. Turbulence is notoriously challenging, in part due to its multiscale nature and the fact that large-scale coherent structures cannot be disentangled from small-scale fluctuations. This closure problem is emblematic of a greater challenge in complex systems, where coarse-graining and statistical mechanics descriptions break down. This work demonstrates an alternative data-driven modeling approach to learn nonlinear models of the coherent structures, approximating turbulent fluctuations as state-dependent stochastic forcing. We demonstrate this approach on a high–Reynolds number turbulent wake experiment, showing that our model reproduces empirical power spectra and probability distributions. The model is interpretable, providing insights into the physical mechanisms underlying the symmetry-breaking behavior in the wake. This work suggests a path toward low-dimensional models of globally unstable turbulent flows from experimental measurements, with broad implications for other multiscale systems.

## Teaser

A low-dimensional data-driven model captures the dominant dynamics in a turbulent flow.

## INTRODUCTION

Despite being nominally deterministic, turbulent flows are characterized by multiscale spatiotemporal chaos. Many traditional analyses have therefore relied on statistical descriptions (*1*). However, it is now known that many inhomogeneous flows are dominated by energetic coherent structures at large scales and low frequencies relative to Kolmogorov’s universal equilibrium range (*2*–*4*). By leveraging this intrinsic structure, reduced-order models of turbulent flows promise to advance engineering objectives in design, optimization, and control (*5*–*7*). However, balancing accuracy and efficiency by simultaneously modeling the evolution of the large-scale structures while accounting for the effects of incoherent fluctuations has been notoriously challenging, especially in a noninvasive fashion that is suitable for experimental measurements.

This modeling challenge stems from the multiscale nature of turbulence. In contrast to systems that can be treated by classical statistical mechanics, there is no strict separation of scales between low-frequency coherent dynamics and turbulent fluctuations. Governing equations for filtered or averaged variables can be derived, but the influence of the unresolved scales cannot be eliminated from the coarse-grained equations, leading to the closure problem. Numerous deterministic closure models have been proposed in the context of high-dimensional Reynolds-averaged Navier-Stokes (RANS) or large eddy simulation (LES) methods (*8*), although these approaches are still high dimensional and computationally expensive.

Alternatively, to take advantage of persistent, energetic coherent structures, semiempirical projection-based methods can be used to derive a compact approximation to the dynamics in the form of amplitude equations governing the evolution of global modes. For example, perhaps the most widely used reduced-order modeling method in fluid dynamics is Galerkin projection of the Navier-Stokes equations onto a modal basis (*2*, *4*, *9*). However, because projection-based methods act as a spatial filter, they face the same closure problems as the RANS and LES methods. Projection methods also require access to both a high-fidelity numerical solver and information about the flow that is difficult to obtain experimentally.

To address the analytic challenges of turbulence modeling, data-driven methods have long played a role in modal analysis (*10*) and reduced-order modeling (*5*, *7*). Recent advances in machine learning have generated increased interest in data-driven methods for fluid dynamics (*11*–*14*), including for turbulence modeling (*15*–*17*) and forecasting (*18*). Despite the expressive power of modern machine learning methods, it is challenging to develop models that are robust, generalizable, and interpretable. The sparse identification of nonlinear dynamics (SINDy) framework (*19*) has promise for interpretable low-dimensional modeling, and it is able to incorporate partial physical knowledge including symmetries, conservation laws, and invariant manifold structure (*20*–*22*). SINDy has mainly been applied to laminar flows with the exception of recent work developing RANS closure models (*23*). Following these successes, our objective is to model coherent structure dynamics with a SINDy-type approach, approximating turbulent fluctuations as noise acting on a few global integral quantities.

## RESULTS

In this work, we develop a sparse nonlinear model of a fully turbulent wake experiment. Our model takes the form

$$\overset{\cdot }{x}=f(x)+\sigma (x)w(t)$$ (1)

where *x* is a generalized state vector and *w*(*t*) is Gaussian white noise. Whereas the RANS and LES models use a relatively high-dimensional discretization of the flow field, here we assume that *x* represents a small set of modal coefficients, order parameters, or other integral quantities capturing relevant large-scale structure in the flow. Moreover, this description in terms of deterministic drift dynamics *f*(*x*) forced by diffusion σ(*x*) is a fundamentally different approach to turbulence modeling. The RANS and LES methods seek a local, deterministic closure model for the effects of the unresolved scales in terms of resolved variables, Eq. 1 approximates the global, statistical influence of turbulent fluctuations on the state *x*.

**Fig. 1.**
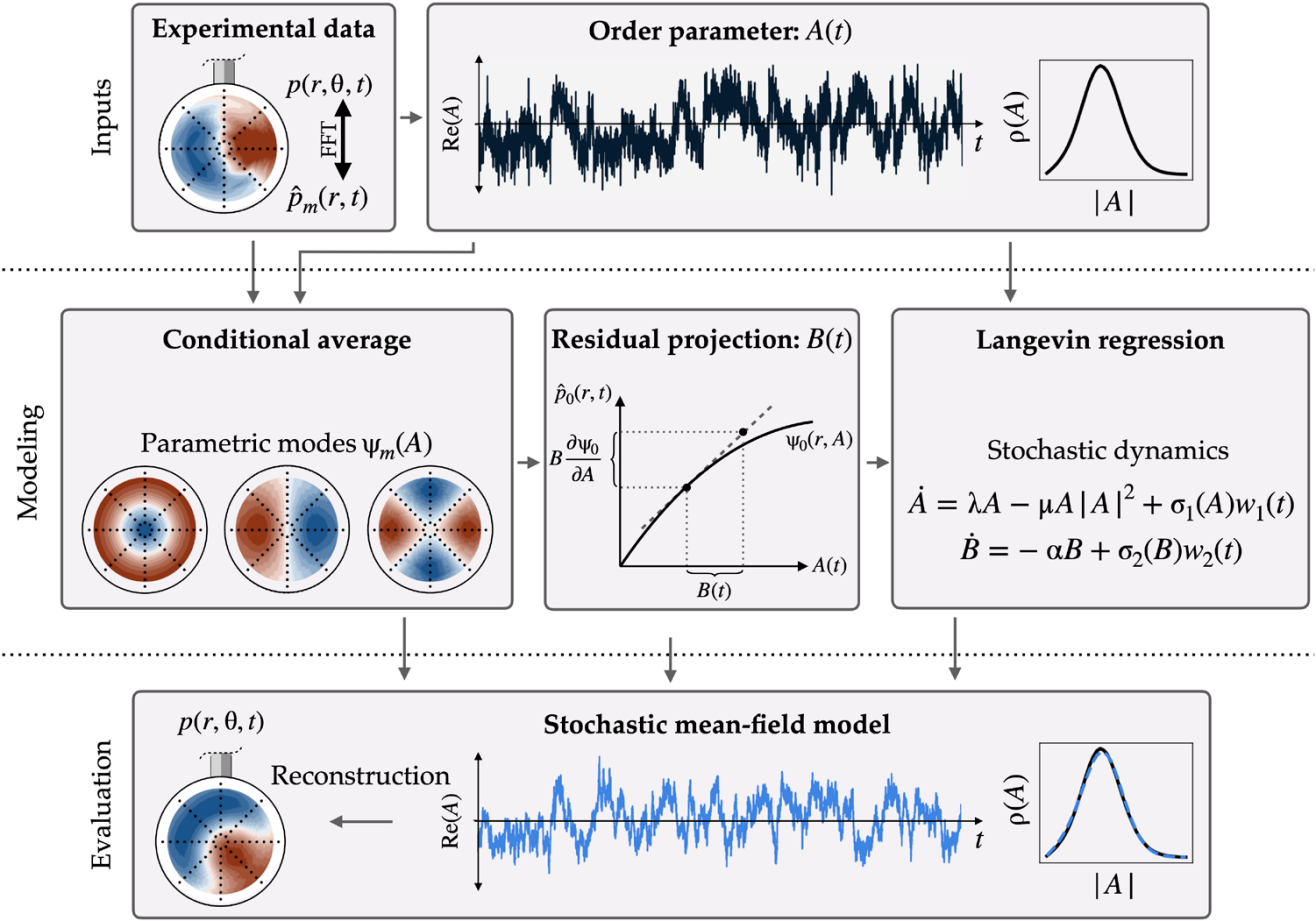
Overview of the model development. Beginning with an order parameter *A*(*t*) computed from the base pressure measurements, the spatial modes ψ*_m_* are computed at each wave number *m* by conditional averaging. These modes define an approximate slow manifold ψ(*A*) that captures the dominant antisymmetric behavior. To fully resolve the symmetric fluctuations, we introduce a generalized shift mode with amplitude *B*(*t*), defined by projection onto the tangent space of this manifold. Last, we identify a nonlinear stochastic dynamical system model with Langevin regression. We can compare the statistics, including the empirical probability distribution ρ, of the model to the experiment with Monte Carlo evaluation of the stochastic system. Because the proposed model (bottom row) is random in nature, it can only reproduce the experimental data (top row) in a statistical sense; in general, neither the time series nor the pressure distributions will match on a point-by-point basis.

We learn the model in Eq. 1 using a recently developed stochastic extension to SINDy, called Langevin regression (see Fig. 1), which is suitable for multiscale systems (*24*, *25*). Progress in model discovery has advanced our ability to approximate stochastic dynamics from limited experimental data (*24*–*31*), even if the model structure is a priori unknown (*24*, *25*, *32*). These methods extend traditional stochastic modeling beyond near-equilibrium systems, providing a useful approximation despite the absence of a strict scale separation.

We demonstrate this approach to model the turbulent wake behind an axisymmetric bluff body, as shown in the section ‘Symmetry-breaking in the bluff body wake’. We reduce the dimension of the experimental measurements by modal decomposition. Traditional methods decompose the field into a fixed set of spatial modes with time-varying amplitudes. The Fourier basis is a convenient representation for coordinates with translational or rotational symmetry. In the present case, the flow is rotationally symmetric, so azimuthal variations can be expanded with Fourier modes *e*^*im*θ^.

**Fig. 2.**
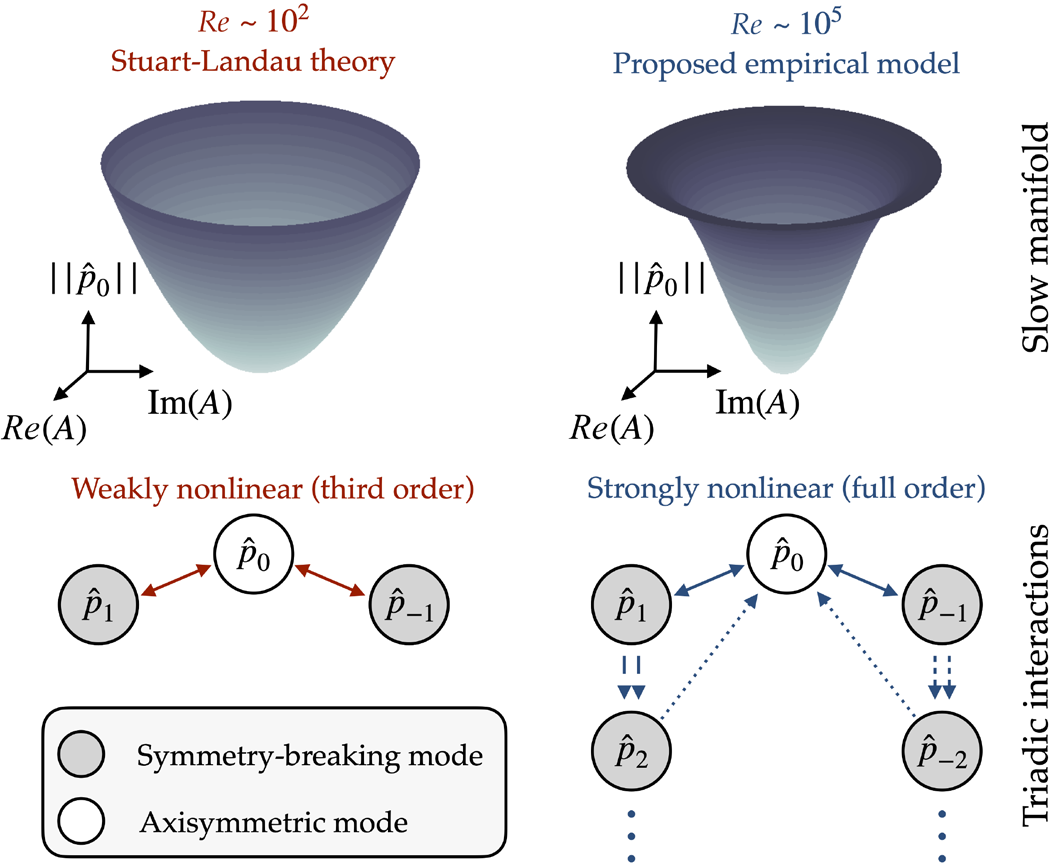
Visualization of approximate slow manifolds and the nonlinear interactions responsible for generating them. Weakly nonlinear analyses of laminar flows typically neglect higher-order interactions for small fluctuations, leading to a parabolic manifold for the low-dimensional dynamics (left). Using conditional averaging, we show that these interactions are necessary to explain observations of the turbulent wake (right). Pairs of lines with similar styles indicate the structure of the leading-order triadic interactions. The physical interactions are between velocity components, but here we use the base pressure field as a proxy for coherent structures in the wake.

Analysis of the Navier-Stokes equations in the wave number domain reveals that the nonlinear advective term only admits triadic interactions in which both the forced and forcing modes have wave numbers that sum to zero. In particular, the axisymmetric fluctuations at *m* = 0 are driven by the “self-interaction” of the complex-conjugate components at *m* = ± 1, ± 2, etc., as shown in Fig. 2. The full nonlinear flow is made up of a complicated network of such interactions across all scales, making it notoriously difficult to construct simplified models with reduced degrees of freedom.

One approach to circumventing this problem, typically near the threshold of some instability, is to perform an asymptotic multiscale expansion and assume that higher-order nonlinear interactions may be neglected. This approximation can resolve the leading-order mean flow deformation because of the self-interaction of the instability mode, a central feature of the Stuart-Landau nonlinear stability mechanism (*33*, *34*). The triadic interactions are truncated at leading order (Fig. 2). In this weakly nonlinear regime, the mean flow deformation can be treated as another fixed spatial mode with an amplitude that depends on the strength of the instability (*9*, *35*, *36*).

However, these tools are largely theoretical and numerical; here, the flow is fully turbulent and thus strongly nonlinear. To avoid the assumption of weak nonlinearity and explore the spatial structure of the mean flow deformation, we estimate the modes from measurements of the base pressure distribution by reducing the symmetry via phase alignment and averaging conditioned on the center of pressure. The amplitude dependence of the symmetric mode deviates significantly from the polynomial scaling predicted by weakly nonlinear analysis, confirming the need for a parametric basis to capture the effect of strongly nonlinear interactions.

Following the conditional average, the method proceeds as shown schematically in Fig. 1. The modal expansion yields physically meaningful order parameters *A*(*t*) and *B*(*t*) related to symmetry-breaking and mean-field deformation in the wake, respectively. We apply Langevin regression to identify an interpretable dynamical system that models the broadband turbulence as stochastic forcing of the low-dimensional symmetry-breaking dynamics. The resulting model is a stochastic Stuart-Landau equation similar to those proposed in previous studies of similar configurations (*28*, *37*–*39*), but with the addition of state-dependent forcing and an additional degree of freedom. Monte Carlo evaluation of the Langevin system compares favorably to the experimental statistics, and the model provides physical insights into low-frequency fluctuations of the axisymmetric recirculation bubble. Each of these will be investigated below.

### Symmetry-breaking in the bluff body wake

**Fig. 3.**
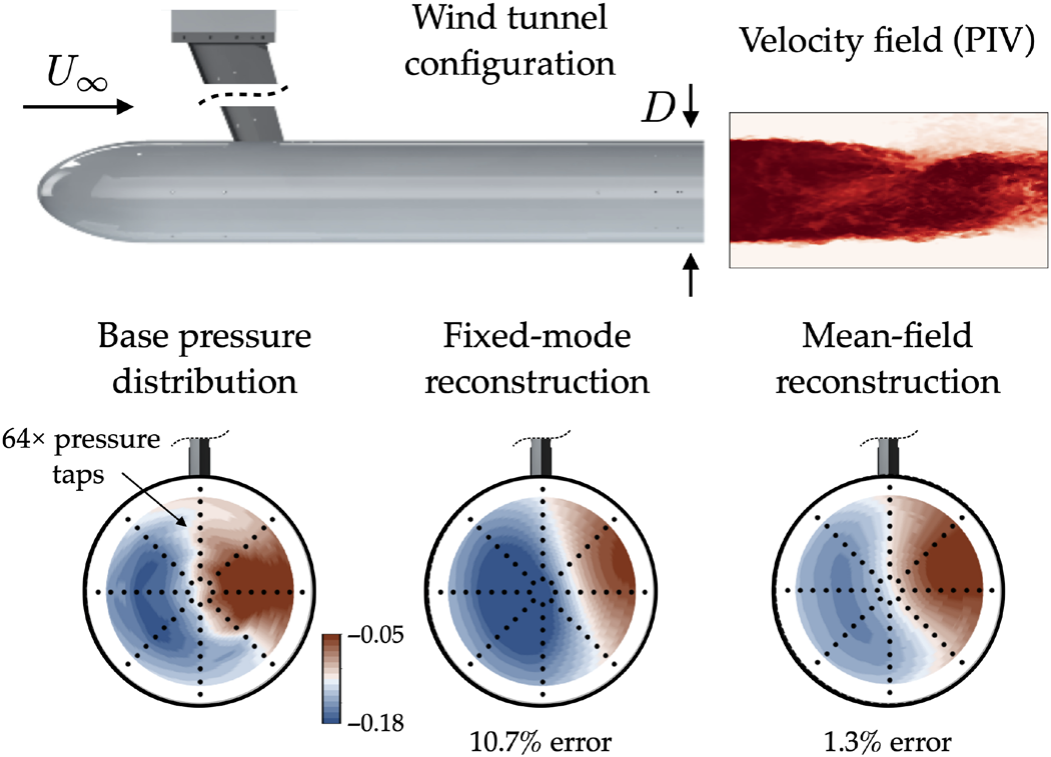
Experimental configuration for the axisymmetric wake. The signature of the dominant symmetry-breaking instability is captured by the complex order parameter defined in Eq. 5. The coherent fluctuations in the pressure distribution can be approximated with the mean-field model (bottom). The proposed model substantially improves the reconstruction over a traditional fixed-mode decomposition. Although not used for the analysis in this work, a particle image velocimetry (PIV) estimate of the velocity magnitude is also shown to visualize the asymmetry and vortex shedding in the wake.

The turbulent wake in Fig. 3 provides a rich test system for model discovery. Flow with free-stream velocity *U*_∞_ and kinematic viscosity ν is deflected around a cylindrical blunt-nosed body with diameter *D*. The spatiotemporal dynamics of the wake are determined by the Reynolds number *Re* = *DU*_∞_/ν. Flows past bluff bodies at low Reynolds number often exhibit stereotypical global instabilities such as von Kàrmàn vortex streets or steady symmetry-breaking wake deflection (*3*, *9*, *36*, *40*, *41*). In the laminar regime, nonlinear saturation of the exponential growth of these instabilities can often be described by Stuart-Landau theory, in which the fluctuations deform the mean flow in a stabilizing feedback loop (*33*, *34*).

The amplitude *A*(*t*) of the instability mode is governed by the cubic Stuart-Landau equation

$$\frac{\mathrm{dA}}{\mathrm{dt}}=(\lambda -\mu {|A|}^{2})A$$ (2)

The term in parentheses is the effective eigenvalue of the instability mode, as modified by the mean flow deformation. When the real part of λ is positive, small perturbations grow exponentially until the instantaneous growth rate reaches a balance with μ∣*A*∣^2^.

Global modes that are qualitatively similar to the laminar instabilities appear to dominate these flows well into the turbulent regime (*42*–*44*). Although the Stuart-Landau mechanism is typically derived via an asymptotic weakly nonlinear analysis, the resulting amplitude equations are often assumed to approximately describe the evolution of turbulent coherent structures as well (*28*, *29*, *37*–*39*).

At very low Reynolds number, the wake is steady, axisymmetric, and laminar. The flow undergoes two bifurcations, becoming linearly unstable to a steady symmetry-breaking mode (${\text{Re}}_{c}^{1}\approx 424$) and a second pair of unsteady vortex-shedding modes (${\text{Re}}_{c}^{2}\approx 605$), both with azimuthal wave number *m* = ± 1 (*44*). By approximating these as a single codimension-2 bifurcation, the weakly supercritical flow can be approximated with an asymptotic expansion and normal form dynamics (*36*). These instability modes continue to dominate the coherent part of the flow even in fully developed turbulence, as has been shown for the present experimental data at *Re* ≈ 2 × 10^5^ (*43*). The base pressure distribution is measured from a set of 64 equally spaced pressure taps from which a time series of 8.9 × 10^6^ measurements are sampled at 225 Hz; further details are given in (*43*). Equally spacing the pressure taps allows us to take advantage of the geometric symmetry with a Fourier series representation of the pressure field. Hence, the sensor distribution determines the wave number resolution of the analysis. In particular, eight sensor stations restrict the Fourier representation to wave numbers *m* = ± 3, although we find that only *m* = 0, ± 1, ± 2 are strongly correlated with the symmetry breaking.

Although the time series of measurements appears stochastic at this Reynolds number, the flow is characterized by semiregular energetic structures, including vortex shedding and a symmetry-breaking wake deflection (*3*, *42*, *43*). These structures can be directly linked to the instability modes and weakly nonlinear dynamics of the corresponding laminar flow (*36*, *45*). Although the vortex shedding is dynamically important in the wake, it is only weakly observable from the pressure signal on the bluff body itself. This also suggests that the vortex shedding is potentially less important to practical drag reduction than the symmetry-breaking wake deflection. In this work, we therefore restrict our attention to the steady symmetry-breaking instability.

This wake deflection is particularly important as it represents generic spatial symmetry-breaking behavior that occurs in a wide variety of three-dimensional bluff body wakes (*28*, *36*, *40*). Moreover, this symmetry breaking is associated with increased pressure drag, making it the target of a variety of active flow control investigations (*38*, *39*, *41*, *45*, *46*). In several of these studies, reduced-order models have played a key role in designing and understanding the actuated system.

### Mean-field theory of symmetry-breaking transitions

The Stuart-Landau nonlinear stability theory is typically derived as an asymptotic expansion in multiple time scales. It therefore falls into the category of weakly nonlinear analysis and is only strictly applicable near the threshold of instability, although the effect is generally understood to persist much beyond the asymptotic regime in many cases (*9*, *47*, *48*). However, Landau also considered another limiting case of dynamics: symmetry-breaking phase transitions of systems in thermodynamic equilibrium. Turbulence is both strongly nonlinear and far from thermodynamic equilibrium, but the observation that both limiting regimes can be described with similar equations motivates the development of phenomenological Stuart-Landau–type models for symmetry-breaking behavior in the turbulent axisymmetric wake.

A generic system in thermodynamic equilibrium that undergoes a continuous symmetry-breaking phase transition at critical temperature *T*_c_ (or temperature analog such as Reynolds number) has an effective potential *V*(*T*, *A*), which we assume can be expanded in the magnitude of a (generally complex) order parameter *A*. A canonical example of this is the Ising model, in which the statistically symmetric disorder of the high-temperature system is broken in a phase transition to a ferromagnetic state below a critical temperature. On the basis of physical symmetries, the effective potential can be expanded as

$$V(T)=V_{0}(T)+V_{1}(T){|A|}^{2}+V_{2}(T){|A|}^{4}+\ldots$$ (3)

This order parameter, which here we assume to be small when suitably nondimensionalized, quantifies the degree of symmetry breaking in the system. The equilibrium condition A_*_ at a given temperature is determined by the minimum free energy with respect to ∣*A*∣. To leading order, $A_{*}(T)=\sqrt{-V_{1}(T)/2V_{2}(T)}.$

For the high–Reynolds number axisymmetric wake studied in this work, the unsteady aerodynamic center of pressure serves as an order parameter capturing the symmetry-breaking wake deflection. When nondimensionalized by the body diameter, its value is small even far from the bifurcation with mean value $\bar{A}\approx 0.032$.

The system is unsteady but statistically stationary. In the thermodynamic perspective, the instantaneous field is disturbed from the minimum potential state by broadband turbulence. We model this as near-equilibrium thermal fluctuations in overdamped Langevin dynamics (*49*)

$$\frac{\mathrm{dA}}{\mathrm{dt}}=-\nabla V(A)+\Sigma (A)w(t)$$ (4)

where *w*(*t*) is a white noise process and Σ is the diffusion function. Expanding *V*(*A*) in ∣*A*∣ to third order, the Langevin model would take the form of the Stuart-Landau equation (2) forced by white noise. Although this qualitative symmetry-based argument suggests the expected structure of the dynamics, we use the recently proposed Langevin regression method for identifying nonlinear stochastic models (*25*) to identify the drift and diffusion functions rather than presuppose this form, as described below.

### Parametric modal expansion

Reduced-order models such as Eq. 1 rely on dominant low-dimensional structure to approximate salient features of the flow with a small set of variables; such low dimensionality in fluid flows often arises as a consequence of global instability. The instability mode responsible for the steady wake deflection in the laminar flow stabilizes at finite amplitude because of mean flow deformation, with temporal evolution given by compact normal form dynamics (*36*). The turbulent wake exhibits qualitatively similar behavior; recent studies have modeled the aerodynamic center of pressure with stochastic Stuart-Landau equations (*28*, *37*). However, while the relationship between the amplitude equations and spatial mean flow deformation is clear for the weakly nonlinear laminar case, it has been unexplored in the phenomenological models developed for turbulent flows.

Standard model reduction methods decompose the field into a set of spatial modes (*10*) with coefficients whose time evolution is governed by the amplitude equations. Within this framework, one way to resolve mean flow deformation is with the addition of a spatial mode parameterizing the difference between the unstable steady state and the mean flow. This additional shift mode can either be derived empirically (*9*) or as a natural product of an asymptotic expansion (*35*). In either case, the assumption of weakly nonlinear interactions implies polynomial scaling of the deformation amplitude with respect to the amplitude of the dominant instability. This approach has proven successful in low-dimensional models of a variety of laminar flows (*20*, *22*, *36*, *47*). However, in strongly nonlinear turbulent flows, this fixed modal basis cannot generally be expected to resolve the mean flow deformation.

To describe the symmetry breaking and associated mean-field deformation, we model the evolution of the base pressure distribution *p*(*r*, θ, *t*), where *r* and θ are polar coordinates on the circular bluff body base. Although the pressure is not a dynamic variable in incompressible flow, the base pressure can be used as a convenient and experimentally accessible proxy for the energetic coherent structures in the wake, because these limited observations can be connected to previously observed wake structures based on symmetries and spectral energy content (*43*). The radial coordinate of the unsteady aerodynamic center of pressure is also a natural order parameter for the degree of symmetry breaking.

Let *p*^0^(*r*) be the pressure associated with the unstable axisymmetric steady state, which is unknown and experimentally inaccessible, and $\bar{p}(r)$ be the temporal mean field estimated from the pressure taps. The self-interaction of the velocity fluctuations associated with *p*′ deforms the unstable steady state to the mean flow via the Reynolds stresses (*8*).

We define the unsteady aerodynamic center of pressure on the bluff body base as a complex-valued order parameter *A*(*t*)

$$A(t)=\frac{1}{\int p(r,\theta ,t)\text{rdrd}\theta }\int p(r,\theta ,t)re^{i\theta }\mathrm{drd}\theta$$ (5)

The amplitude ∣*A*(*t*)∣ is a measure of the degree of asymmetry in the wake, while its phase gives the azimuthal orientation of the wake deflection. We approximate this integral with a Riemann sum over the 64 pressure sensor locations.

The instantaneous strength of the coherent antisymmetric fluctuations associated with ∣*A*(*t*)∣ is responsible for the axisymmetric mean flow deformation and nonlinear equilibrium of the instability mode (*36*). The mean-field $\bar{p}$ and steady-state *p*^0^ are therefore associated with the mean amplitude $\bar{A}\equiv \bar{|A(t)|}$ and *A* = 0, respectively, although both fields are themselves axisymmetric. Similarly, an instantaneous amplitude ∣*A*(*t*)∣ between 0 and $\bar{A}$ is associated with an axisymmetric field interpolating between *p*^0^(*r*) and $\bar{p}(r)$, although the amplitude itself only directly represents antisymmetric fluctuations. In other words, the part of the instantaneous *m* = 0 field that resolves the Stuart-Landau deformation mechanism is a direct function of the order parameter. More broadly, we expect that the part of the field that is coherent with the order parameter can be revealed with an average conditioned on its amplitude.

### Coherent fields via conditional averaging

On the basis of the symmetry of the flow, we expand the pressure field with a Fourier series $p(r,\theta ,t)=\sum_{m}{\hat{p}}_{m}(r,t)e^{\mathrm{im}\theta }$. The preceding discussion suggests that the part of the field that is coherent with the symmetry breaking might be approximated with a parametric modal decomposition ${\hat{p}}_{m}(r,t)\approx \psi_{m}(r,A(t))$. In contrast to a fixed space-time separation of variables, such as is used in proper orthogonal decomposition or dynamic mode decomposition, this approach allows the modes to naturally deform with the instantaneous order parameter amplitude. Once the modes ψ*_m_*(*r*, *A*) are known, such a representation reduces the field to a function of this single complex degree of freedom.

For example, if the self-interaction of the fluctuations are assumed to be weakly nonlinear and higher-order nonlinearity is neglected, then the axisymmetric component can be approximated with the leading term in a polynomial expansion, i.e., ψ_0_(*r*, *A*) = *p*^0^(*r*) + ∣*A*∣^2^*p*_Δ_(*r*) + O(∣*A*∣^4^). In a numerical setting, the “shift mode” *p*_Δ_(*r*) can be determined either through an asymptotic expansion about the unstable base flow (*35*, *36*) or from the difference between the base and mean flows (*9*).

**Fig. 4.**
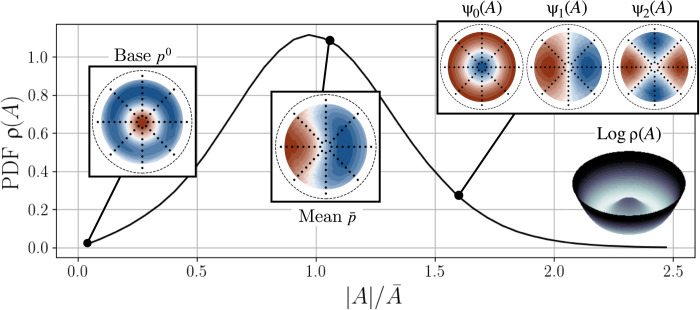
Illustration of phase-aligned conditional averaging. We assume that the average at small amplitudes is representative of the pressure distribution associated with the unstable steady state *q^B^*. The field at any other point can be approximated with spline interpolation, allowing us to explore the amplitude dependence of the coherent fields. Also shown for reference are the unconditional phase-aligned mean field and the estimated two-dimensional log-probability density function (PDF), which is roughly analogous to a potential field.

Experimentally, neither of these approaches is viable, because the unstable steady state is typically unavailable. Instead, we propose identifying the parametric modes with a phase-aligned conditional average on the order parameter, visualized in Fig. 4. This procedure, described in detail in Materials and Methods, estimates the part of the field at each wave number that is correlated with the order parameter amplitude ∣*A*(*t*)∣. A continuous estimate of the modes can then be constructed with a spline interpolation of the conditional averages ψ*_m_*(*r*,∣*A_i_*∣).

**Fig. 5.**
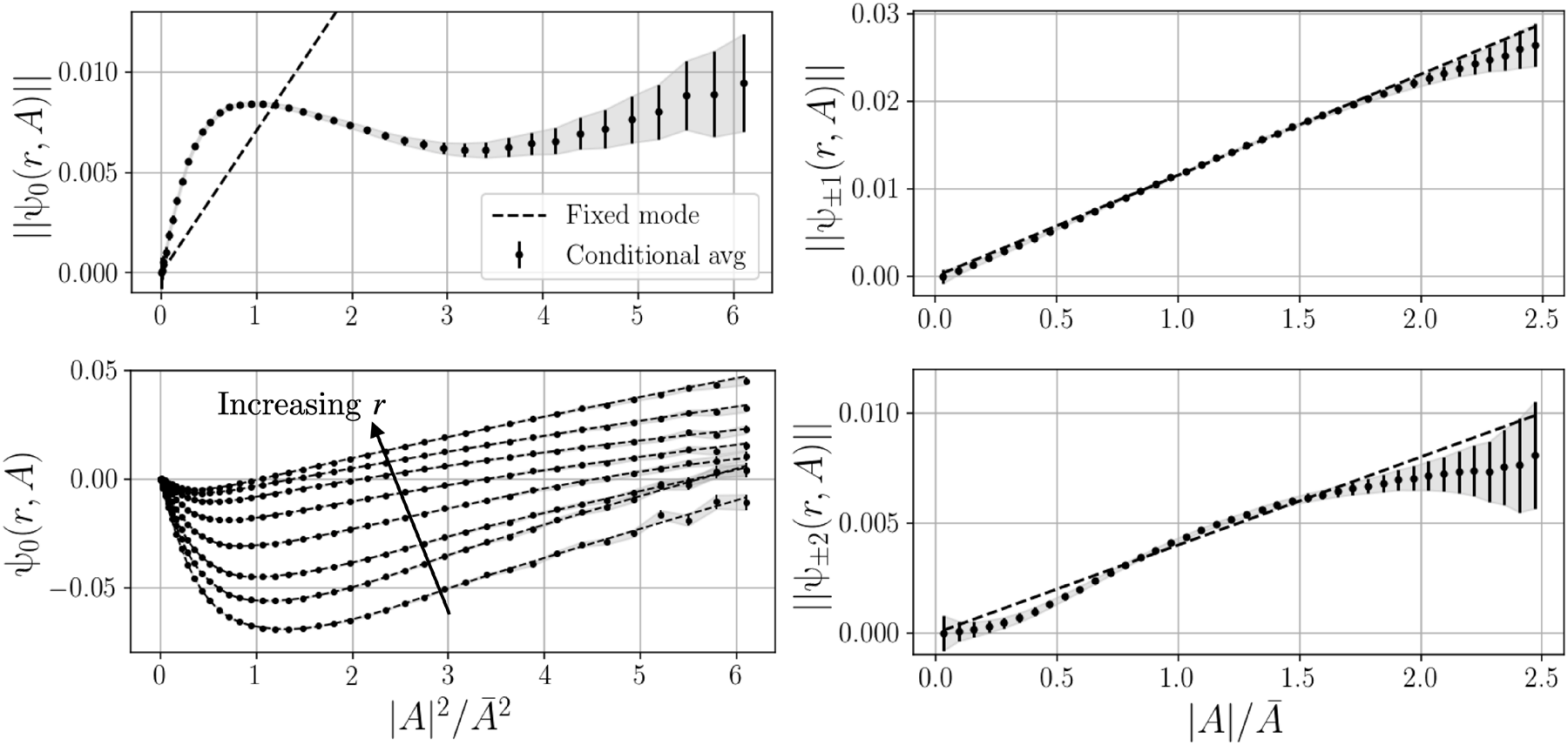
Field deformation in the conditional average. The phase-aligned average gives the coherent fields as a function of instability amplitude ∣*A*∣ for various azimuthal wave numbers *m*. The deformation of the axisymmetric part of the field cannot be explained by a single fixed mode or weakly nonlinear scaling (**A**), although the deformation has a smooth dependence on the amplitude (**C**). We approximate this amplitude dependence with a spline fit (C, dashed lines). On the other hand, the symmetry-breaking fields at *m* = ± 1, ± 2 are consistent with the fixed-mode assumption of Stuart-Landau theory (**B** and **D**).

Figure 5 shows the radially integrated modes, along with the axisymmetric mode at each radial sensor location. The conditional average is compared to a fixed mode approximation where the spatial structure is fixed at its value at the mean amplitude $\bar{A}$. We draw several conclusions from the amplitude scaling of these modes. First, the axisymmetric field at *m* = 0 cannot be described by a fixed mode. The weakly nonlinear scaling ψ_0_ ∼ ∣*A*∣^2^ does not hold for typical amplitudes in this case. Figure 5C also shows the value of the *m* = 0 field at each of the eight radial sensor locations as a function of ∣*A*∣^2^; a single fixed mode cannot explain this behavior even if its integrated amplitude is a complicated function of ∣*A*∣. This indicates that the nonlinear axisymmetric self-interaction and higher antisymmetric harmonics play an important role in altering the spatial structure of the axisymmetric deformation as the fluctuation amplitude changes. However, for a good approximation, both the *m* = ±1 and *m* = ±2 Fourier components can be reasonably well described by a single fixed mode with linear dependence on ∣*A*∣. This is consistent with the typical assumption of the Stuart-Landau theory that the instability is a fixed eigenmode of the linear operator with variable eigenvalue. Higher wave numbers show weak coherence with the order parameter; we therefore truncate the reconstruction at ∣*m*∣ = 2.

The conditional average can be viewed as an empirical approximation of the slow manifold related to the symmetry-breaking behavior. Figure 2 visualizes this manifold by revolving the radially integrated axisymmetric deformation about the $\left\|{\hat{p}}_{0}\right\|$ axis, while the parabolic “theoretical” manifold is generated from the weakly nonlinear ∣*A*∣^2^ scaling.

This conditional averaging approach can be viewed as an empirical approximation to the self-consistent mean-field model (*48*), because it gives the fields at arbitrary fluctuation amplitudes without assuming a fixed spatial structure as in standard modal analyses. In contrast to the self-consistent model, however, we do not neglect the influence of higher harmonics on the base flow. Because the modes are derived directly from experimental data, they naturally account for nonlinear interactions at all orders and can resolve arbitrary deformations of the axisymmetric component.

### Residual projection for mean flow modification

Because the symmetry breaking is linked to an instability mode, we expect that antisymmetric modes will not significantly deform with amplitude, so ψ*_m_*(*r*,∣*A*(*t*)∣) ≈ ∣*A*(*t*)∣ψ*_m_*(*r*) for *m* ≠ 0. This is confirmed by the conditional averaging analysis above. However, the coherent part of the axisymmetric component is dominated by nonlinear deformations induced by Reynolds stresses, so it may have a complicated amplitude dependence.

For weakly nonlinear laminar flows, an asymptotic analysis indicates that axisymmetric deformations can be expressed as a function of the instability amplitude ∣*A*(*t*)∣^2^, pinning the state to the slow manifold (*35*, *36*). However, the structure of models based on Galerkin projection onto a set of fixed spatial modes suggests that there may be some finite relaxation time to the equilibrium state determined by ∣*A*(*t*)∣ (*9*).

In other words, the conditional average defines an approximate slow manifold (illustrated in Fig. 2), although we would like to avoid assuming the state always resides on this surface. We address this by introducing an additional (real) degree of freedom *B*(*t*) representing the difference between the axisymmetric field ${\hat{p}}_{0}(r,t)$ and the manifold field ψ_0_(*r*, ∣*A*(*t*)∣^2^). If this difference is typically small, then the axisymmetric field can be expressed as a linearization about ∣*A*(*t*)∣ by defining a new *m* = 0 mode ψ*_B_*(*r*, ∣*A*(*t*)∣^2^) from the gradient of the manifold

$${\hat{p}}_{0}(r,A,B)\approx p^{0}(r)+\psi_{0}(r,{|A|}^{2})+2|A|B\psi_{B}(r,{|A|}^{2})$$ (6A)

$$\psi_{B}(r,{|A|}^{2})={\frac{\partial \psi_{0}}{\partial {|A|}^{2}}|}_{{|A(t)|}^{2}}$$ (6B)

This model for the axisymmetric part of the field is a generalization of the fixed shift mode model proposed by (*9*). In particular, if the axisymmetric field does have the weakly nonlinear scaling ψ_0_(*r*, ∣*A*(*t*)∣^2^) = ∣*A*(*t*)∣^2^ψ_0_(*r*) and the linearization is about the unstable fixed point *A* = 0, then the tangent field in Eq. 6 is just ψ_0_(*r*). For models based on Galerkin projection, it is more natural to define a single coefficient for each mode. That is, in the models introduced by (*9*) and (*29*), *B* is defined so that ${\hat{p}}_{0}(r,B(t))\approx B(t)\psi_{0}(r)$. However, the proposed parametric expansion in the present work allows the model more flexibility to capture the natural variations of the flow, without assuming any scaling behavior. Further details and intuition for the proposed model of axisymmetric fluctuations are given in the Supplementary Materials, including a visualization of the residual projection.

The instantaneous value of this secondary order parameter *B*(*t*) can be estimated by projecting the part of the axisymmetric field not correlated with ∣*A*∣ onto the tangent space of the slow manifold

$$B(t)\approx \frac{\int ({\hat{p}}_{0}(t)-\psi_{0}(t))\psi_{B}(t)\mathrm{rdr}}{\int \psi_{B}(t)\psi_{B}(t)\mathrm{rdr}}$$ (7)

where the explicit dependence on *r* and ∣*A*(*t*)∣ has been omitted everywhere for notational clarity. In practice, if the axisymmetric mode ψ_0_(*r*, ∣*A*(*t*)∣^2^) is interpolated in ∣*A*∣^2^ with a spline, then the tangent field ψ*_B_*(*r*, ∣*A*(*t*)∣^2^) can be computed with a derivative of the spline at each radial position *r*.

We emphasize that the conditional averaging has already resolved the axisymmetric deformations associated with the Stuart-Landau nonlinear stability mechanism. This secondary order parameter *B*(*t*) is therefore not strictly necessary to describe the symmetry-breaking behavior, but it significantly improves the resolution of the model for the axisymmetric fluctuations, reducing the error in the modal approximation by 37%. Moreover, as shown below, the new degree of freedom *B*(*t*) that allows for deviations from the slow manifold introduces the a finite relaxation time scale that can account for a previously unexplained peak in the symmetric power spectrum.

With this modal expansion, the base pressure field can be reconstructed with

$$p(r,\theta ,t)=\underset{\begin{array}{c} \text{axisymmetric}\\ +\underset{\text{antisymmetric}}{\underbrace{|A|\sum_{m}\psi_{m}(r)e^{\mathrm{im}(\theta +\phi (t))}}} \end{array}}{\underbrace{p^{0}(r)+\psi_{0}(r,{|A|}^{2})+2|A|B\psi_{B}(r,{|A|}^{2})}}$$ (8)

where the time dependence of *A*(*t*) and *B*(*t*) has been omitted for brevity. The full model consists of seven modes: the unstable steady state *p*^0^, the symmetric deformation ψ_0_, the linearization of the manifold ψ*_B_*, and the instability mode at each wave number *m* = ± 1, ± 2. However, the model only has three degrees of freedom: the real and imaginary parts of the complex instability amplitude *A*(*t*) and the real axisymmetric deviation from the manifold *B*(*t*). The modal expansion (8) has a straightforward dependence on these degrees of freedom, so it will behave predictably even when evaluated for a state not in the original dataset. All of the modes, along with their parametric variation in *A*, can be directly computed from experimental observations. An example reconstructed field is shown in Fig. 3.

### Reduced-order model of symmetry breaking

We identify a stochastic model of the form of Eq. 1 by applying Langevin regression to the generalized modal coefficients *A*(*t*) and *B*(*t*), approximating the incoherent turbulent fluctuations as Gaussian white noise. This method optimizes unknown parameters of a proposed nonlinear stochastic model using forward and adjoint Fokker-Planck equations to enforce consistency with experimental statistics. If the form of the model is unknown, then a simple reverse-greedy model selection procedure can identify the simplest model that explains the observed data (*25*). This method explicitly treats inherent fluctuations of the system as process noise. We explore its sensitivity to measurement noise in the Supplementary Materials and find that it is robust to well-behaved noise up to a signal-to-noise ratio of around 1.

Reduced-order models of turbulent flows are generally challenging to construct for the same reason that the statistical perspective was so successful historically; signals are dominated by apparently random turbulent fluctuations. The most common approach to dealing with the fluctuations is to introduce a closure model that approximately captures their effects, for instance, via an eddy viscosity term to achieve the correct dissipation, without explicitly treating the fast scales (*2*, *4*, *50*).

A major difficulty in treating the fast fluctuations directly is that there is not a strict enough separation of scales to apply the machinery of near-equilibrium statistical mechanics. Although they cannot be formally justified, in practice some approximations based on statistical mechanics have been successful. For example, while Eulerian statistics are usually non-Gaussian, central limit theorem arguments suggest that both Lagrangian variables and integral quantities have near-normal distributions (*1*, *51*). Similarly, although the turbulent fluctuations are correlated in time, various modeling methods have, nevertheless, been successful by approximating them as white noise (*37*, *52*, *53*). Alternatively, recent work has investigated the use of colored noise in linearized flow models (*54*, *55*), although classical statistical physics tools such as the Fokker-Planck equation, which is integral to our system identification method, cannot be applied in this case.

In previous work, the evolution of the order parameter magnitude ∣*A*(*t*)∣ has been successfully modeled by a stochastic cubic amplitude equation, inspired by the weakly nonlinear normal form (*37*). However, the weakly nonlinear analysis is predicated on a fixed-mode decomposition, which is at odds with the proposed amplitude-dependent spatial modes. Nevertheless, as described above, a dynamical model with similar structure can also arise from the mean-field theory of symmetry-breaking phase transitions, which does not rely on fixed spatial modes or weak nonlinearity.

### Model analysis and evaluation

**Fig. 6.**
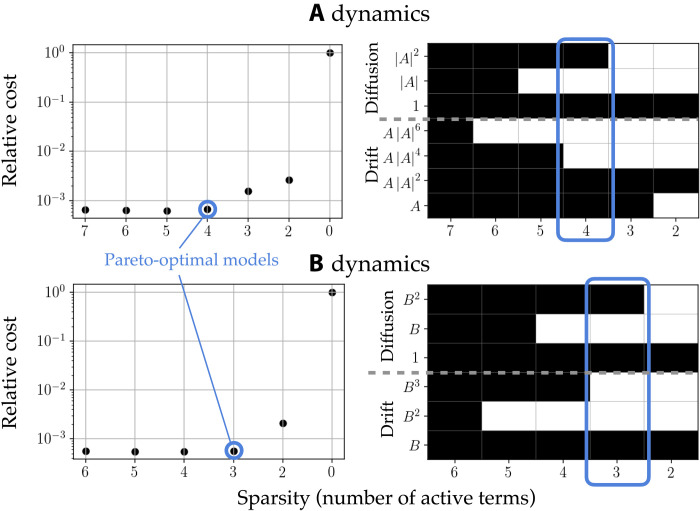
Model selection with Langevin regression. The drift and diffusion functions are sparse linear combinations from a library of candidates. We select the simplest models that are statistically consistent (small cost function). The vertical axes are scaled by the value of the cost function with an identically zero coefficient vector.

Langevin regression identifies clear Pareto-optimal models for both the *A* and *B* dynamics, as shown in Fig. 6. That is, we can select a model with minimal complexity in the sense that neglecting any more terms would cause the optimization loss function to significantly increase. The full identified model is

$$\overset{\cdot }{A}=\lambda A-\mu A{|A|}^{2}+(\sigma_{A}+\gamma_{A}{|A|}^{2})w_{1}(t)$$ (9A)

$$\overset{\cdot }{B}=-\alpha B+(\sigma_{B}+\gamma_{B}B^{2})w_{2}(t)$$ (9B)

with coefficients given in Table 1.

**Table 1.** Identified coefficients for the Langevin models (9).

**λ**	**μ**	**σ*_A_***	**γ*_A_***	**α**	**σ*_B_***	**γ*_B_***
1.9	−1.9	0.8 + 0.7*i*	0.3 + 0.3*i*	−26.7	6.7	0.2

The drift function for the order parameter *A* identified by the model selection has the form of a Langevin-Stuart-Landau equation, while the drift of the axisymmetric deformation *B* is linear relaxation. Physically, because *B* is defined as the difference between the instantaneous axisymmetric component of the field and that given by the conditional average on ∣*A*∣, this models noisy relaxation toward the location on the slow manifold defined by the instantaneous value of *A*. This is consistent with the Fourier-decomposed Navier-Stokes equations; the axisymmetric field does not instantaneously reach the equilibrium corresponding to the amplitude of the antisymmetric instability, although the relaxation time scale is often considered negligible in weakly nonlinear laminar flows (*9*, *36*).

The method also identifies quadratic state-dependent diffusion for both *A* and *B*. This is perhaps unexpected, because the turbulent fluctuations are expected to be locally isotropic and therefore approximately independent of the large-scale motions (*8*). However, similar state-dependent diffusion arises in the case that slow macroscopic dynamics are averaged over fast degrees of freedom that are excited by stochastic forcing due to nonlinear coupling across scales (*52*, *56*). In particular, the diffusion functions in Eq. 9 have the same form as a Taylor expansion of the diffusion derived by (*56*) for an unstable mode with quadratic coupling to stable modes driven by additive white noise. The state-dependent diffusion also allows the Langevin model to better resolve the long tails of the probability distributions, as previously observed by (*25*).

To evaluate the model, we simulate the Langevin models and construct approximate pressure fields using the parametric modal expansion (6). This reconstruction returns from the three-dimensional state space of the Langevin model to the 64-dimensional space of the pressure measurements. We compare the full base pressure distributions to test the ability of the entire model, including both Eqs. 8 and 9, to capture the physical behavior.

**Fig. 7.**
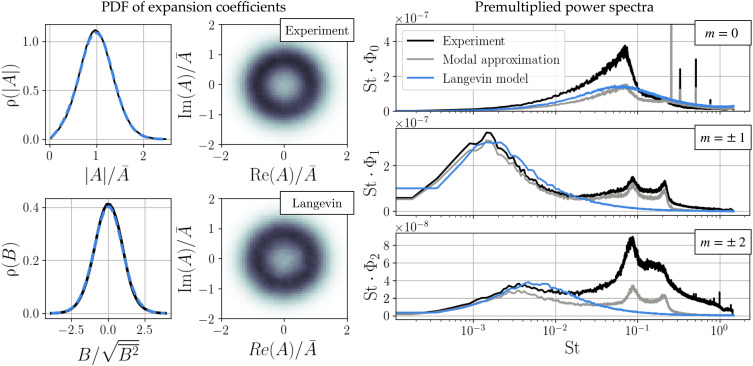
Statistical evaluation of the model. The mean-field modal expansion accurately captures most features of the radially averaged premultiplied power spectrum based only on the order parameters *A*(*t*) and *B*(t) (gray). Monte Carlo evaluation of the Langevin model (blue) shows that it reproduces the empirical probability distributions and dominant frequency content at the leading azimuthal wave numbers.

The results are compared to both the experimental data and the reconstruction based on the modal expansion and experimental values of *A* and *B* in Fig. 7 using both the empirical probability distribution and the radially averaged premultiplied power spectra. We compare the premultiplied spectra St · Φ*_m_*(St), where Φ*_m_*(St) is the estimated power spectral density of the radially integrated *m*th Fourier component, rather than the power spectral density itself because the area under the premultiplied spectrum directly corresponds to signal energy. This makes it better suited for comparison of the dominant energy scales. Although the model is too simplified to capture the full power spectrum, it does reproduce the dominant peak for the leading wave numbers and accurately approximates the probability distributions.

The compact empirical model (Eq. 9) therefore resolves the dominant physical mechanisms associated with symmetry breaking in the turbulent wake, including linear instability, mean-field deformation, and the influence of higher harmonics. Because Eq. 9 gives the drift and diffusion in terms of simple analytic expressions, the behavior of the model can be fully characterized throughout the low-dimensional state space. For example, the Fokker-Planck equation can be used to either numerically compute the steady-state probability density or predict the evolution of an uncertain distribution of states. Thus, although the model is fit to a finite dataset, its behavior in “unseen” regions (e.g., $|A|\gg \bar{A}$) can be completely quantified.

Critically, the model also reproduces the dominant frequency peak in the axisymmetric power spectrum, previously described as a “bubble-pumping mode” (*3*, *28*, *43*), although it does not appear in any stability analysis (*44*). This model suggests that this spectral peak may instead be viewed as the result of a finite relaxation time scale toward the axisymmetric deformation induced by the amplitude of the instability mode. The spatial field associated with these dynamics is the gradient of the manifold defined by the conditional average. These relaxation dynamics are consistent with the underlying Navier-Stokes equations, but this time scale is often considered negligible for weakly nonlinear laminar flows.

## DISCUSSION

We have demonstrated a data-driven modeling approach to learning interpretable nonlinear models for fluid coherent structures, where multiscale turbulence is treated as state-dependent stochastic forcing. This approach has been used to develop a simplified mean-field model of the symmetry-breaking behavior in a turbulent axisymmetric wake. The empirical model comprises seven parametrically varying spatial modes and three dynamical degrees of freedom and was constructed entirely from experimental observations. Using a phase-aligned conditional average with respect to the center of pressure, we have shown that the fixed-mode ansatz of standard modal decompositions cannot explain the mean-field deformation related to the symmetry-breaking instability of the turbulent axisymmetric wake.

This reflects the higher-order influences and nonlinear self-interaction of the axisymmetric part of the flow, both of which are typically negligible for weakly supercritical laminar flows.

Modeling approaches based on weakly nonlinear approximations have proven highly successful in laminar flows. However, in this work, we have shown that the extension of this perspective to turbulent flows is more subtle than adding stochastic forcing to the weakly nonlinear model. The amplitude scaling and structure of the axisymmetric deformation are inconsistent with the quadratic dependence implied by such an approach, although the proposed conditional averaging procedure can capture the natural behavior of the mean flow deformation.

Langevin regression identifies a simple Stuart-Landau–type model for the modal coefficients and a quadratic nonlinear state-dependent noise model. This form of diffusion is consistent with analysis of fast-slow systems with quadratic nonlinearities where only the fast scales are stochastically forced (*52*, *56*). Monte Carlo evaluation of the model matches the stationary probability distributions of the experimental data and resolves the dominant peak in the power spectrum at the leading azimuthal wave numbers. Moreover, the model is simple and interpretable, yielding physical insights into physical mechanisms including the mean flow deformation and low-frequency modulation of the recirculation bubble.

Beyond the context of the axisymmetric wake, these results support the parameterization of turbulent fluctuations as stochastic forcing of the quasi-deterministic coherent structures evolving near a slow manifold, at least as an approximation for empirical models. We emphasize that this description relies on a strict separation of scales, which is known to be absent in turbulent flows. Even with this caveat, this simplification is appealing enough for engineering applications such as closed-loop flow control that it may be useful even if it only holds in an approximate sense. For example, a feedback controller based on a nonlinear Langevin model has been shown to produce power-efficient drag reduction on an Ahmed body with a similar symmetry-breaking instability (*38*).

More broadly, we hope that this work illustrates a principled approach to constructing stochastic reduced-order models from limited experimental observations of a turbulent flow. Although much progress has recently been made in developing stochastic models of turbulent flows using the linearized governing equations (*54*, *55*) and with empirical models of experimental data (*29*, *37*, *39*), there are many open questions about how the heuristics of low-dimensional models of weakly nonlinear flows will translate to fully developed turbulence. In this work, we have chiefly focused on the mean-field deformation associated with the symmetry-breaking bifurcation, but recent studies have highlighted the role of higher-order triadic nonlinear interactions in capturing the dynamics of both natural (*57*) and actuated (*39*) turbulent wakes. Capturing the interactions between instability modes (for instance, symmetry breaking and vortex shedding) may also prove critical in developing models suitable for active flow control.

As fully empirical data-driven methods continue to grow in popularity and utility, ensuring consistency with the underlying physics remains a central challenge. The model proposed in this work captures many of the essential statistical features of the data and leads to previously unknown hypotheses about the behavior of the axisymmetric wake in particular and globally unstable turbulent flows in general.

## MATERIALS AND METHODS

### Phase-aligned conditional average

We expect that the higher-order nonlinear interactions in the turbulent wake may lead to more complicated amplitude dependence compared to the weakly nonlinear laminar regime. We therefore propose identifying parametric modes with a phase-aligned conditional average on the order parameter. The phase alignment reduces the symmetry of the fields; without this step, all asymmetry would vanish on averaging. On the other hand, the conditional average captures the natural variation of the field with the order parameter amplitude without any assumptions on the functional form of the *A* dependence.

Beginning with the Fourier decomposition into modes ${\hat{p}}_{m}(r,t)$, we compute the order parameter with Eq. 5 in amplitude-phase representation *A* = ∣ *A* ∣ *e*^*i*ϕ^. The phase of the order parameter can then be removed from each field

$$p\prime (r,\theta ,t)=\sum_{m}{\hat{p}}_{m}(r,t)e^{\mathrm{im}(\theta -\phi (t))}=\sum_{m}{\hat{p}\prime }_{m}(r,t)e^{\mathrm{im}\theta }$$ (10)

We divide the space of observed order parameter amplitudes ∣*A*∣ into histogram bins centered on *A_i_* with width 2Δ*A*. For each wave number *m* and histogram bin *i*, the radial component of ψ*_m_*(*r*, *A_i_*) is approximated with

$$\psi_{m}(r,|A_{i}|)=\langle {\hat{p}\prime }_{m}(r,t)|\|A(t)|-|A_{i}\|<\Delta A\rangle$$ (11)

We also estimate the base field *p*^0^(*r*) as the conditional mean at *m* = 0 for the smallest histogram, i.e., over fields for which ∣*A*(*t*) ∣ < Δ*A*.

### Nonlinear stochastic system identification

We use the recently proposed Langevin regression method for identifying nonlinear stochastic models (*25*) to identify the drift and diffusion functions rather than presuppose a form based on qualitative arguments. Although we opt for this method because it enables identification of a statistically consistent low-dimensional nonlinear generalized Langevin equation of the form (1), other system identification methods could also be used for this step. For example, in the Supplementary Materials, we compare Langevin regression to a model constructed with vector autoregression, a popular method in time series forecasting.

Langevin regression, a stochastic variant of the SINDy approach (*19*), optimizes free parameters of a Langevin model via solutions of both the forward and adjoint Fokker-Planck equations. The steady-state solution of the forward equation gives the steady-state probability distribution, while the adjoint solution gives the finite-time propagation statistics of the Langevin model (*49*, *58*). Langevin regression simultaneously minimizes the discrepancy between the Fokker-Planck solutions and statistics computed from the experimental data. This system identification approach does not require access to the governing equations and can be applied to arbitrary quantities of interest. Details about the algorithm and numerical methods for solving the Fokker-Planck equations are given in (*59*) and the Supplementary Materials.

Moreover, this approach can be combined with the simple reverse-greedy stepwise sparse regression procedure for selecting a parsimonious but maximally descriptive model from a set of candidates (*24*, *25*). This algorithm sequentially discards functions whose exclusion is associated with the smallest increase in cost function. The Pareto-optimal model can then be chosen as the simplest model with a small cost function, as shown in Fig. 6. We use a library of polynomials consistent with the expected symmetries as candidate functions. The drift and diffusion functions are approximated by sparse linear combinations of these functions. For instance, the drift and diffusion libraries for *A* are

$$\Theta_{f}^{A}(A)=[A\;A{|A|}^{2}\;A{|A|}^{4}\;A{|A|}^{6}]$$ (12A)

$$\Theta_{\sigma }^{A}(A)=[\begin{array}{ccc} 1 & |A| & {|A|}^{2} \end{array}]$$ (12B)

Then, the approximate drift function is $f_{A}(A)=\Theta_{f}^{A}(A)\xi_{f}^{A}$, where $\xi_{f}^{A}$ is a relatively sparse coefficient vector that identifies the library terms that are active in the dynamics. We do not include the axisymmetric deviation amplitude *B* because it is not associated with any symmetry breaking in the flow and therefore would not appear in the effective potential for *A*. The rotational symmetry of the physical system implies that $\xi_{f}^{A}$ should be purely real, but because Langevin regression is based on a least-squares method, a small imaginary part will generally be retained. For the sake of a minimum-complexity model, we enforce that $\xi_{f}^{A}$ is real, although allowing complex-valued coefficients does not noticeably change the results.

Likewise, the libraries for *B* are

$$\Theta_{f}^{B}(B)=[\begin{array}{ccc} B & B^{2} & B^{3} \end{array}]$$ (13A)

$$\Theta_{\sigma }^{B}(B)=[\begin{array}{ccc} 1 & B & B^{2} \end{array}]$$ (13B)

Again, we do not expect strong coupling between the order parameters *A* and *B*, because *B* was defined primarily to capture fluctuations that were uncorrelated with *A*.

Decoupling the dynamics also reduces the maximum dimensionality of the regression problems from three to two dimensions, because the real and imaginary parts of *A* must be treated separately. Because Langevin regression is a histogram-based method where the Fokker-Planck equation is solved and evaluated on a grid, it does not scale well to higher dimensions. For problems with multiple coupled instability modes, histogram-free approaches such as Langevin inference (*30*, *60*) may be more appropriate, although this does not enforce consistency with the steady-state probability distribution.

The two key parameters in this method are the finite sampling rate, which allows the fast turbulent fluctuations to appear approximately uncorrelated in the time series, and the weight of the Kullback-Leibler (KL) divergence between the empirical probability distribution and the steady-state solution of the Fokker-Planck equation. The sensitivity of the results to these choices and some criteria for making this selection are given in the Supplementary Materials. The latter controls the relative weight factor of the forward and adjoint Fokker-Planck solutions in the objective function. We choose sampling rates 200 times slower than the experimental sampling rate for *A* and 50 times slower for *B* and select the KL divergence weight so that the forward and adjoint Fokker-Planck solutions have approximately equal contributions in the optimization. The values are 10^−1^ and 10^2^ for the *A* and *B* optimizations, respectively.

Because Langevin regression identifies by fitting the statistics of the experimental data, it depends fundamentally on statistical convergence of the dataset. We verify this in the Supplementary Materials and show that the variation in the mean and root mean square of the pressure measurements converge to∼1% with around 1% of the data, while the model coefficients reach 1% convergence with 50% of the data.
