# Supplementary material for: An empirical mean-field model of symmetry-breaking in a turbulent wake
Source: Sci Adv. 2022 May 11;8(19):eabm4786. doi: 10.1126/sciadv.abm4786 (PMC9094670; doi:10.1126/sciadv.abm4786)
Supplement: Supplementary file 1 — Supplementary text S1 to S7 Figs. S1 to S7 Tables S1 and S2 [file sciadv.abm4786_sm.pdf]

Science Advances
AAAS

# Supplementary Materials for

## An empirical mean-field model of symmetry-breaking in a turbulent wake

Jared L. Callaham*, Georgios Rigas, Jean-Christophe Loiseau, Steven L. Brunton

*Corresponding author. Email: jc244@uw.edu

Published 11 May 2022, *Sci. Adv.* **8**, eabm4786 (2022)
DOI: 10.1126/sciadv.abm4786

**This PDF file includes:**

Supplementary text S1 to S7
Figs. S1 to S7
Tables S1 and S2

# Supplementary Materials

## S1 Axisymmetric mean flow modification

While previous work has explored the use of a stochastic model to approximate the dynamics of a global integral quantity (*37*), one contribution of the present work is the parametric modal decomposition, which seeks to connect this low-dimensional approximation to the symmetry-breaking in the flow field. A critical part of this model, which departs from otherwise similar models of laminar flow (*9, 35, 36*), is our treatment of axisymmetric fluctuations related to the mean flow modification. In this section we expand on the rationale and methodology for this component of the mean field model.

**Mean flow deformation** A central challenge in the study of turbulence is that the average flow field, which is the quantity of practical engineering interest, is not generally a steady-state solution of the Navier-Stokes equations. The dimensionless Navier-Stokes equations governing incompressible fluid flow are

$$\frac{\partial \boldsymbol{u}}{\partial t} + (\boldsymbol{u} \cdot \nabla)\boldsymbol{u} = -\nabla p + \frac{1}{\mathrm{Re}}\nabla^2 \boldsymbol{u}, \tag{S1a}$$

$$\nabla \cdot \boldsymbol{u} = 0. \tag{S1b}$$

Here the Reynolds number $\mathrm{Re} = DU_\infty/\nu$ is based on free-stream velocity $U_\infty$, kinematic viscosity $\nu$, and length scale $D$ (in this case the diameter of the bluff body). We may collect the linear and quadratic parts of Eq. (S1) and express it in operator form as a single equation in $\boldsymbol{q} = [\boldsymbol{u}\, p]^T$

$$\frac{\partial \boldsymbol{q}}{\partial t} = \mathcal{N}(\boldsymbol{q}) = \mathcal{L}(\boldsymbol{q}) + \mathcal{Q}(\boldsymbol{q}, \boldsymbol{q}). \tag{S2}$$

A steady-state "base flow" thus $\boldsymbol{q}_b(\boldsymbol{x})$ solves

$$0 = \mathcal{N}(\boldsymbol{q}_b). \tag{S3}$$

The stability of this solution is determined by the fate of perturbations $\boldsymbol{q}'$, so that

$$\boldsymbol{q}(\boldsymbol{x}, t) = \boldsymbol{q}_b(\boldsymbol{x}) + \boldsymbol{q}'(\boldsymbol{x}, t). \tag{S4}$$

When the perturbations $\boldsymbol{q}'$ are sufficiently small the quadratic interaction $\mathcal{Q}(\boldsymbol{q}', \boldsymbol{q}')$ can be ignored and the problem is linear:

$$\frac{\partial \boldsymbol{q}'}{\partial t} = \mathcal{L}(\boldsymbol{q}') + \mathcal{Q}(\boldsymbol{q}_b, \boldsymbol{q}') + \mathcal{Q}(\boldsymbol{q}', \boldsymbol{q}_b) \equiv \mathcal{L}_{\boldsymbol{q}_b}(\boldsymbol{q}'). \tag{S5}$$

Numerically and analytically, this can be approached as an eigenvalue problem in terms of eigenvalues $\lambda_k$ and associated eigenvectors $\boldsymbol{q}_k$:

$$\lambda_k \boldsymbol{q}_k = \mathcal{L}_{\boldsymbol{q}_b}(\boldsymbol{q}_k) \tag{S6}$$

At high Reynolds numbers the flow is likely to be linearly unstable ($\mathrm{Re}(\lambda_k) > 0$ for at least one $\lambda_k$), in which case $\boldsymbol{q}_b$ is unlikely to be naturally observed and is mainly of theoretical importance. However, global eigenmodes from Reynolds numbers much closer to the threshold of instability can still be relevant in the fully nonlinear, turbulent flow. For example, the primary laminar instability modes of the axisymmetric wake are steady symmetry-breaking and periodic vortex shedding (*36*), both of which persist as coherent structures in the fully turbulent wake (*43, 44*).

Practically, a more relevant quantity than the unstable base flow is the mean flow $\bar{\boldsymbol{q}}(\boldsymbol{x})$. We can no longer assume typical perturbations are small, but the field can still be decomposed into the mean and fluctuations:

$$\boldsymbol{q}(\boldsymbol{x}, t) = \bar{\boldsymbol{q}}(\boldsymbol{x}) + \boldsymbol{q}'(\boldsymbol{x}, t). \tag{S7}$$

Substituting into Eq. (S2) and averaging (with $\overline{q'} = 0$ by definition), the mean flow is a solution to the *Reynolds-averaged Navier-Stokes* (RANS) equations

$$0 = \mathcal{N}(\bar{\boldsymbol{q}}) + \overline{\mathcal{Q}(\boldsymbol{q}', \boldsymbol{q}')}. \tag{S8}$$

Comparing to Eq. (S3), the mean flow is "deformed" by the Reynolds stress term $\overline{\mathcal{Q}(\boldsymbol{q}', \boldsymbol{q}')}$, describing the mean self-interaction of the fluctuations.

While Eq. (S3) can be solved numerically with large-scale iterative root-finding methods, Eq. (S8) is not *closed* in that there is no equation determining $\boldsymbol{q}'$. This leads to the central closure problem of turbulence; much of the computational field of turbulence modeling revolves around accurately approximating the effect of $\overline{\mathcal{Q}(\boldsymbol{q}', \boldsymbol{q}')}$ without resorting to extensive direct numerical simulation.

In the experimental context the problem is inverted; the mean can be computed simply by averaging a long time series, while the operators $\mathcal{L}, \mathcal{N}$, etc. are not accessible. Here we make the standard assumption that the flow is statistically stationary and invoke ergodicity to approximate the ensemble average with its temporal counterpart. However, we still seek to understand and model the physics associated with the mean flow and dominant unsteady coherent structures. We use the term "mean field theory" in this context to refer to the analysis of the generation and sustainment of the mean flow, especially by dominant instability modes of the flow (*47, 48*).

**Self-consistent model** One instructive numerical method for closing Eq. (S8) is the *self-consistent* mean flow model (*48*). This method assumes that the flow was dominated by a single instability mode $\boldsymbol{q}_1$, with associated leading eigenvalue $\lambda_1$. In particular, the method builds on the "real zero imaginary frequency" heuristic, which assumes both that the mean is marginally stable and that the imaginary part of the leading eigenmode of the flow linearized about the *mean* (rather than the steady state) is a good approximation of the dominant frequency of the nonlinear flow.

The self-consistent model constructs a closed model as follows. Subtracting the RANS equations (S8) from the full Navier-Stokes equations (S2),

$$\frac{\partial \boldsymbol{q}'}{\partial t} = \mathcal{L}_{\bar{\boldsymbol{q}}}(\boldsymbol{q}') + \mathcal{Q}(\boldsymbol{q}', \boldsymbol{q}') - \overline{\mathcal{Q}(\boldsymbol{q}', \boldsymbol{q}')}. \tag{S9}$$

Approximating the perturbation with the normal mode ansatz $\boldsymbol{q}'(\boldsymbol{x}, t) = A\boldsymbol{q}_1(\boldsymbol{x})e^{i\lambda_1 t} + \text{c.c.}$ for some complex constant $A$, the RANS and perturbation equations (S8) and (S9) become the coupled quasisteady system

$$\lambda_1 \boldsymbol{q}_1 = \mathcal{L}_{\bar{\boldsymbol{q}}}(\boldsymbol{q}_1) \tag{S10a}$$

$$\mathcal{N}(\bar{\boldsymbol{q}}) = -|A|^2 \overline{\mathcal{Q}(\boldsymbol{q}_1^*, \boldsymbol{q}_1)} + \text{c.c.}. \tag{S10b}$$

In the self-consistent method, these equations are solved in tandem for increasing values of $|A|$ until $\text{Re}(\lambda_1) = 0$. Physically, the method treats the self-interaction $\overline{\mathcal{Q}(\boldsymbol{q}_1^*, \boldsymbol{q}_1)}$ as a forcing term approximating the Reynolds stresses. Eq. (S10) can be interpreted as a closed-loop system; the Navier-Stokes operator linearized about the mean flow generates the perturbation, while the self-interaction of the perturbation in turn deforms the mean flow. The two are in balance when the predicted growth rate $\text{Re}(\lambda_1) = 0$, indicating the mean flow is marginally stable.

**Approximation with conditional averaging** Although the self-consistent modeling procedure clearly cannot be applied to experimental data, The parametric modal decomposition proposed in this work aims to emulate the self-consistent description of the nonlinear equilibrium between the fluctuations and mean flow. In particular, the self-consistent model solutions for $\bar{\boldsymbol{q}}$ and $\boldsymbol{q}_1$ can be viewed as parametric modes that depend on the fluctuation amplitude $|A|$. Here we expand on this intuition and describe the conditional averaging used as an empirical approximation of the self-consistent fields.

Based on the axial symmetry of the flow and sensor locations, the base pressure distribution $p(r, \theta, t)$ can be expanded with Fourier modes

$$p(r, \theta, t) = \sum_m \hat{p}_m(r, t)e^{im\theta}. \tag{S11}$$

The dominant symmetry-breaking behavior occurs at the antisymmetric wavenumbers $m = \pm 1$ (*36*). The symmetry-breaking can be quantified with the $(r, \theta)$ coordinates of the unsteady

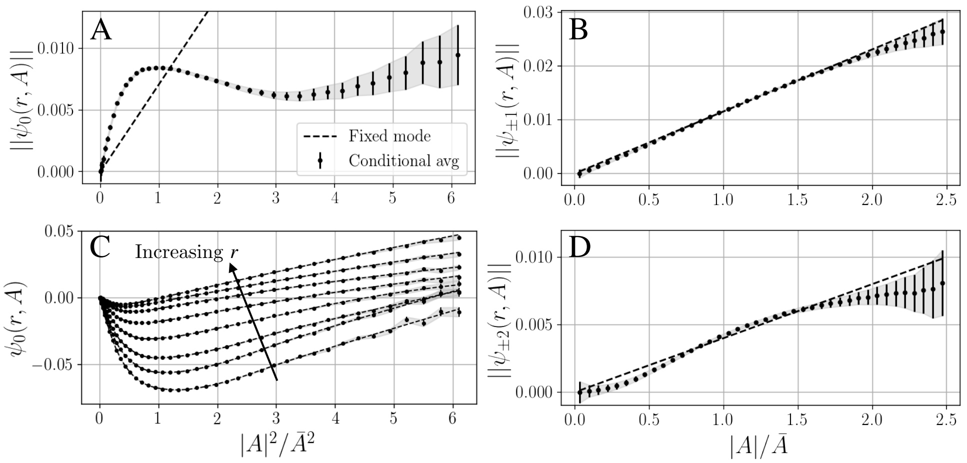


**Supplemental Material, Figure S1: Field deformation in the conditional average.** The scaling predicted by weakly nonlinear analysis holds for antisymmetric fluctuations (**B**, **D**), but breaks down for the axisymmetric component (**A**). The axisymmetric fluctuations cannot be captured by a single fixed mode (**C**), so instead we treat it as a parametric function of amplitude $|A|^2$ and interpolate the conditional average with a univariate spline fit at each radial sensor location (**C**, dashed lines).

aerodynamic center of pressure (*37, 43*), which we express as a complex number $A(t) = |A(t)|e^{i\phi(t)}$. In contrast to standard reduced-order modeling, where a linear modal decomposition defines a set of expansion coefficients used as generalized coordinates, here it is more natural to begin with the low-dimensional quantity and work backwards to the modal basis. Consequently, we assume that part of the field that is coherent with $A$ can be captured with a single parametric mode $\psi_m(r, A(t))$ at each wavenumber.

In the numerical self-consistent model, the amplitude $A$ is fixed as a constant input to the solver and the parametric mode is the solution to Eq. (S10). Experimentally, since $A$ is a fluctuating function of time we approximate the mode with a conditional average. Before doing so, we reduce the symmetry of the fields with a phase alignment:

$$p'(r, \theta, t) = \sum_m \hat{p}_m(r, t)e^{im(\theta - \phi(t))} = \sum_m \hat{p}'_m(r, t)e^{im\theta}. \tag{S12}$$

This step preserves coherent asymmetries in the fields, which would otherwise vanish on averaging over arbitrary phases $\phi$.

We perform the conditional average by dividing the space of observed order parameter amplitudes $|A|$ into histogram bins centered on $A_i$ with width $2\Delta A$. For each wavenumber $m$ and histogram bin $i$, the radial component of $\psi_m(r, A_i)$ is approximated with

$$\psi_m(r, |A_i|) = \left\langle \hat{p}'_m(r, t) \middle| \big||A(t)| - |A_i|\big| < \Delta A \right\rangle_t . \tag{S13}$$

The scaling of the resulting modes are shown in Fig. S1. Again, these may be interpreted in terms of the self-consistent mean field model. The axisymmetric field $\psi_0$ in Fig. S1c is the

average deformation as a function of the fluctuation amplitude $|A(t)|$, analogous to the parametric "mean" $\bar{\boldsymbol{q}}(|A|)$ that solves Eq. (S10) for a given value of $|A|$. We may also approximate the base flow as $p^0(r) \approx \psi_0(r, 0)$, although this is largely notional, as we have no experimental way to verify whether this is associated with a steady-state solution.

Likewise, the antisymmetric fields $\psi_{\pm 1}$, $\psi_{\pm 2}$ are the equivalent of the fluctuations $\boldsymbol{q}_1$ that result in the average deformation $\psi_0$. These amplitude-dependent modes can be easily compared to the fixed spatial modes typically assumed in a model reduction or weakly nonlinear analysis, as shown by dashed lines in Fig. S1. As expected based on the assumption that the symmetry-breaking is linked to a global instability mode, the antisymmetric modes appear to scale linearly with the order parameter amplitude, i.e. $\hat{p}'_m(r,t) \approx |A(t)|\psi_m(r)$ for $m = \pm 1, \ \pm 2$. With eight radial sensor locations we are able to resolve up to $m = \pm 3$, but we truncate at $|m| = 2$ because the conditional average does not yield a significant signal at $|m| = 3$.

On the other hand, while weakly nonlinear analysis predicts that the axisymmetric mode will scale like $\psi_0(r, |A(t)|) \sim |A(t)|^2 \psi_0(r)$, this is clearly not seen in the data (Fig. S1a). The simplest explanation for this is that the two fundamental assumptions leading to the amplitude-squared scaling, namely that the flow is dominated by a single instability mode and that it is characterized by either a timescale separation or weakly nonlinear self-interactions of the fluctuations, are violated in this fully turbulent flow. To respect the strongly nonlinear interactions, we instead model the axisymmetric deformations as an arbitrary function of $|A|^2$, i.e. $\hat{p}_0(r,t) \approx p^0(r) + \psi_0(r, |A(t)|^2)$. We approximate $\psi_0(r, |A(t)|^2)$ by interpolating the conditional average at each radial sensor location and histogram bin $A_i$ with a univariate spline, as shown in Fig. S1c.

Restoring the variable complex phase $\psi(t)$, the pressure field can be approximately reconstructed with the expansion

$$p(r, \theta, t) = p^0(r) + \psi_0(r, |A(t)|) + |A(t)| \sum_{|m|=1,2} \psi_m(r) e^{im(\theta + \phi(t))}. \tag{S14}$$

**Residual projection** In principle, the reconstruction given by the conditionally averaged fields in Eq. (S14) could be enough to describe both the symmetry-breaking behavior and the associated mean flow deformation. However, it turns out that the conditional average does a poor job of approximating the true axisymmetric fluctuations, as shown by the grey reconstruction in Fig. S2.

We propose as a possible explanation for this failure that Eq. (S14) assumes that the *instantaneous* axisymmetric field is given as a function of only $|A|$. This is the natural result of a weakly nonlinear analysis (*33, 35, 36*) or center manifold reduction (*9, 47*), but likely holds only in an average sense for fully developed turbulence. Treating the mean flow deformation as a direct function of $A$ assumes the field is continually residing on the "slow manifold" defined by $\psi_0(r, |A(t)|)$, which will not generally be the case in a turbulent flow. In other words, although $\psi_0(r, |A(t)|)$ is the *mean* deformation associated with the amplitude $|A(t)|$, in general the axisymmetric field may not respond instantaneously to changes in amplitude.

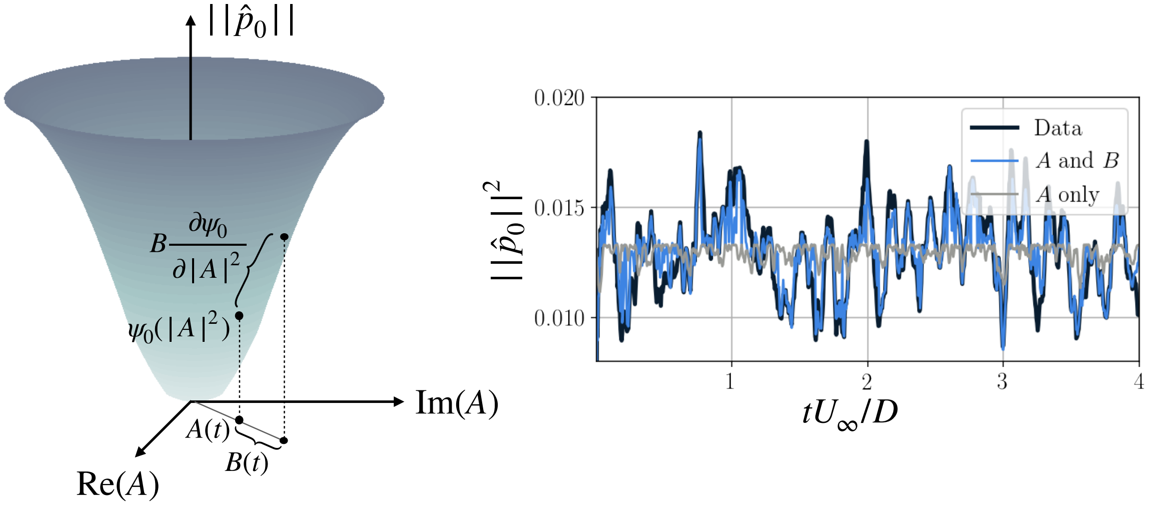


**Supplemental Material, Figure S2:** **Model for axisymmetric fluctuations.** The conditional average on the order parameter $A$ defines a slow manifold (left), but including an additional degree of freedom to account for fluctuations significantly improves the resolution of the $m = 0$ component (right).

To account for these departures from the slow manifold, we introduce a secondary real-valued order parameter $B(t)$ that models the difference between the $\hat{p}_0(r)$ and the reconstruction based on $A$ alone, $p^0(r)+\psi_0(r,|A(t)|^2)$. Assuming this difference is typically small, we expand $\hat{p}$ with a linearization about the instantaneous value of $|A|^2$:

$$\hat{p}_0(r, A(t), B(t)) \approx p^0(r) + \psi_0(r, |A(t)|^2) + 2|A(t)|B(t) \left.\frac{\partial \psi_0}{\partial |A(t)|^2}\right|_{|A(t)|^2}. \tag{S15}$$

The spatially varying component of the final term can be treated as a new parametric mode $\psi_B(r, |A(t)|^2)$ and approximated with a derivative of the spline interpolation of $\psi_0(r, |A|^2)$. Then the full reconstruction based on both order parameters is

$$p(r, \theta, t) \approx \underbrace{p^0(r) + \psi_0(r, |A|^2) + 2|A|B\psi_B(r, |A|^2)}_{\text{axisymmetric}} + \underbrace{|A| \sum_m \psi_m(r) e^{im(\theta+\phi(t))}}_{\text{antisymmetric}}, \tag{S16}$$

where time dependence in $A$ and $B$ has been suppressed for brevity.

While $A(t)$ can be computed directly from the base pressure measurements, the secondary order parameter $B(t)$ is a derived quantity that only has meaning in terms of Eq. (S15). We therefore estimate $B(t)$ with a "residual projection": we project the part of the axisymmetric field not correlated with $|A|$ onto the tangent space of the slow manifold:

$$B(t) \approx \frac{\int \left(\hat{p}_0(r,t) - \psi_0(r, |A(t)|^2)\right) \psi_B(r, |A(t)|^2) r \mathrm{d}r}{\int \psi_B^2(r, |A(t)|^2) r \mathrm{d}r}, \tag{S17}$$

where as usual in this work the spatial integrals are approximated with a Riemann sum. Figure S2 shows that the reconstruction using both $A$ and $B$ in Eq. (S16) significantly improves the approximation of the axisymmetric part of the field compared to using only $A$ in Eq. (S14).

**Relationship to weakly nonlinear analysis** We conclude this section with a discussion of the correspondence between the proposed modal expansion and weakly nonlinear analysis. This presentation is a brief qualitative sketch; the particulars will of course vary depending on flow geometry, symmetries, etc. For a rigorous analysis of the axisymmetric wake see for instance (*36, 45*).

Nonlinear stability theory was introduced by Stuart (*33*) and Landau (*34*) to explore the fate of unstable eigenmodes. The linear theory predicts exponential energy growth, but clearly this soon diverges from reality. In particular, the linear approximation fails once the perturbation reaches finite amplitude and the quadratic interaction $\mathcal{Q}(\boldsymbol{q}', \boldsymbol{q}')$ in the Reynolds decomposition of Eq. (S2) is no longer negligible.

The leading-order correction can be understood most easily for the case discussed in the self-consistent model where the flow is slightly above the threshold of bifurcation so that there is a single complex-conjugate pair of unstable eigenmodes $\boldsymbol{q}_1^{(*)}(\boldsymbol{x})$ with associated eigenvalues $\lambda_1^{(*)} = \gamma_1 \pm i\omega_1$. Weakly nonlinear analysis typically proceeds by representing the solution as an asymptotic expansion assuming both proximity to the bifurcation and a separation in time scales, so that the instability modes have a slowly varying amplitude envelope

$$\boldsymbol{q}'(\boldsymbol{x}, t) = A(\epsilon t)\boldsymbol{q}_1(\boldsymbol{x})e^{i\omega_1 t} + \text{c.c.}, \tag{S18}$$

where $\epsilon \ll 1$ is related to the bifurcation parameter, e.g. $\epsilon \sim \sqrt{(\mathrm{Re} - \mathrm{Re}_c)/\mathrm{Re}_c}$ and the growth rate $\gamma_1$ is $\mathcal{O}(\epsilon^2)$.

At leading order in $\epsilon$ this yields the linear stability problem, which in turn predicts $A \approx e^{\gamma_1 t}$. However, expanding to successive orders gives an explicit model for the closed-loop interactions that generate the mean flow and stabilize the energy of the perturbation, similar to Eq. (S10). Typically the self-interaction of the instability mode contributes a correction to the base flow $\boldsymbol{q}_\delta \sim \boldsymbol{q}_1\boldsymbol{q}_1^*$, so that the "instantaneous" base flow (defined more precisely by averaging over time scales on the order of $\omega_1^{-1}$) is

$$\boldsymbol{q}_0 \approx \boldsymbol{q}_b(\epsilon) + |A|^2\boldsymbol{q}_\delta, \tag{S19}$$

allowing for $\epsilon$ dependence in the base flow.

This in turn modifies the instantaneous growth rate of the envelope amplitude, leading to a Stuart-Landau equation of the form

$$\frac{\mathrm{d}A}{\mathrm{d}t} = \lambda_1 A - \mu A|A|^2. \tag{S20}$$

In general this effect can be stabilizing or destabilizing, but in the common case where it tends to stabilize the base flow, $\mathrm{Re}(\mu) > 0$ and Eq. (S20) describes a self-exciting, self-limiting

nonlinear oscillator. Further from the bifurcation (but still in the laminar regime with a single dominant instability), a similar result can be obtained with an invariant manifold reduction (*9, 47*).

In the axisymmetric wake the analysis is complicated by the fact that there are multiple closely-spaced bifurcations; a steady symmetry-breaking instability followed closely by unsteady vortex shedding, both at wavenumber $m = \pm 1$. However, both instabilities contribute a mean flow modification similar to Eq. (S19) at $m = 0$. In this work we neglect contributions from the vortex shedding, since it is only weakly observable from the base pressure distribution. Hence, the weakly nonlinear analysis predicts that the axisymmetric $m = 0$ deformation will have a fixed spatial structure given by $\boldsymbol{q}_\delta(\boldsymbol{x})$ and will scale with $|A|^2$.

As a final note, while Stuart's derivation and more recent analyses make use of this asymptotic expansion approach, Landau argued the form of Eq. (S20) with a more general qualitative argument that places fewer restrictions on its validity. It is therefore possible, as we find in this work, that Eq. (S20) may be an appropriate amplitude equation even when the flow is strongly nonlinear and Eq. (S19) no longer holds.

# S2 Langevin regression

The details of the Langevin regression algorithm for stochastic system identification are given in (*25*), combining elements from a number of previous works (*24, 26, 27*). Here we give a brief summary for completeness; for a more rigorous treatment of Langevin and Fokker-Planck equations see (*49*).

We assume that the dynamics of a vector-valued observable $\boldsymbol{x}$ (not to be confused with the coordinates $\boldsymbol{x}$ in the previous section) can be approximated with a Langevin equation

$$\dot{x}_i = f_i(\boldsymbol{x}) + \sigma_i(\boldsymbol{x}) w_i(t) \tag{S21}$$

with a deterministic "drift" component $\boldsymbol{f}(\boldsymbol{x})$ and state-dependent diffusion $\boldsymbol{\sigma}(\boldsymbol{x})$ modifying a Gaussian white noise disturbance $\boldsymbol{w}(t)$. Eq. (S21) uses the subscript convention for tensor summation. The stationary probability distribution $p(\boldsymbol{x})$ of $\boldsymbol{x}$ governed by Eq. (S21) is the solution to the steady-state Fokker-Planck equation

$$0 = -\frac{\partial}{\partial x_i}\left[f_i(\boldsymbol{x})p(\boldsymbol{x})\right] + \frac{\partial^2}{\partial x_i \partial x_j}\left[a_{ij}(x)p(x,t)\right], \tag{S22}$$

where $a_{ij} = \sigma_i(\boldsymbol{x})\sigma_j(\boldsymbol{x})/2$. In this work we assume that the diffusion tensor is diagonal, i.e. $a_{ij} = 0$ for $i \neq j$ and $a_{ii} \equiv a_i$. This is not strictly necessary, but significantly simplifies the calculations and we did not find any evidence to contradict the assumption.

One method of identifying approximate Langevin equations from data is to use the Kramers-Moyal average. Defining finite-time conditional moments

$$m_i^{(n)}(\boldsymbol{x}, \tau) = \left\langle \left(x_i'(t+\tau) - x_i'(t)\right)^n \right\rangle_{\boldsymbol{x}'(t)=\boldsymbol{x}}, \tag{S23}$$

the drift and diffusion coefficients are given by the limits

$$f_i(\boldsymbol{x}) = \lim_{\tau \to 0} \frac{1}{\tau} m_i^{(1)}(\boldsymbol{x}, \tau), \qquad a_i(\boldsymbol{x}) = \lim_{\tau \to 0} \frac{1}{2\tau} m_i^{(2)}(\boldsymbol{x}, \tau). \tag{S24}$$

These can be approximated empirically by dividing state space into histogram bins and computing a conditional average of the first and second moments in each bin. However, the sampling rate $\tau$ cannot be taken to zero, nor would it be desirable to do so in practice; Eq. (S24) relies on the assumption of white-in-time process noise $\boldsymbol{w}$. This is not true of multiscale turbulence, so choosing a coarse sampling rate is desirable to allow the fast scales to decorrelate or "whiten" (*25*).

However, both empirical (*26*) and theoretical (***?***) evidence demonstrates that finite-time approximation of the Kramers-Moyal average introduces systematic distortion. In particular, the finite-time conditional moments can be derived from the solution $w_i^{(n)}$ to a particular time-dependent adjoint Fokker-Planck problem:

$$\frac{\partial w_i^{(n)}}{\partial t} = f_j(\boldsymbol{x}) \frac{\partial w_i^{(n)}}{\partial x_j} + a_j(\boldsymbol{x}) \frac{\partial^2 w_i^{(n)}}{\partial x_j^2}, \qquad w_i^{(n)}(\boldsymbol{x}, 0) = x_i^n. \tag{S25}$$

Then the moments are given by

$$m_i^{(1)}(x, \tau) = w^{(1)}(x, \tau) - x \tag{S26a}$$

$$m_i^{(2)}(x, \tau) = w^{(2)}(x, \tau) - 2x w^{(1)}(x, \tau) - x^2, \tag{S26b}$$

from which the drift and diffusion can be computed via Eq. (S24).

As originally proposed by (*27*), this can be used to formulate a parameter optimization problem by minimizing the difference between observed and predicted finite-time conditional moments for a given set of parameters. Langevin regression combines this idea with the stochastic SINDy framework, in which a model is selected from a library of candidate functions (*19, 24*). Defining matrices $\boldsymbol{\Theta}_{f_i}^T(\boldsymbol{x})$ and $\boldsymbol{\Theta}_{\sigma_i}^T(\boldsymbol{x})$ whose columns are the candidate functions evaluated on the discretized domain $\boldsymbol{x}$ and flattened, the drift and diffusion functions are approximated as

$$f_i(x) = \boldsymbol{\Theta}_{f_i}^T(\boldsymbol{x}) \xi_{f_i} \qquad \sigma_i(\boldsymbol{x}) = \boldsymbol{\Theta}_{\sigma_i}^T(\boldsymbol{x}) \xi_{\sigma_i}. \tag{S27}$$

In order to optimize the parameters $\xi$, the domain is first discretized and both the empirical PDF $\hat{p}(\boldsymbol{x})$ and finite-time conditional moments $\hat{m}_i^{(n)}(\boldsymbol{x}, \tau)$ are computed for a specified sampling rate $\tau$ using the discretized domain as histogram bins. Combining all parameters into a single vector $\xi$, the Langevin regression objective function is

$$V(\xi) = \sum_{n=1}^{2} \sum_{i=1}^{d} \boldsymbol{\varepsilon}_i^{(n)^T} W_i^{(n)} \boldsymbol{\varepsilon}_i^{(n)} + \eta \mathcal{D}_{\text{KL}} \left( \hat{p}(\boldsymbol{x}), p(\boldsymbol{x}; \xi) \right), \tag{S28}$$

where $\boldsymbol{\varepsilon}_i^{(n)}(\xi) = m_i^{(n)}(\boldsymbol{x}, \tau; \xi) - \hat{m}_i^{(n)}(\boldsymbol{x}, \tau)$ is the error in the $n^{\text{th}}$ finite-time conditional moment for the $i^{\text{th}}$ variable, $W_i^{(n)}(\boldsymbol{x})$ is a diagonal matrix of weights, $\mathcal{D}_{\text{KL}}$ is the Kullback-Leibler

divergence between the empirical and predicted PDFs, and $\eta$ is a relative weight between the two terms in the objective. For weights, we use the inverse of the standard error in the finite-time conditional mean for each histogram bin. Since computing a gradient of $V$ involves the relatively expensive propagation through the transient PDE (S25) and $\xi$ is relatively low-dimensional, we found it was more computationally efficient to use a gradient-free Nelder-Mead simplex optimizer than either analytic or automatic differentiation. Our source code is available at `github.com/dynamicslab/langevin-regression`.

# S3 Fokker-Planck solver details

Optimizing the Langevin regression cost function $V$ involves repeated solutions of both the steady-state Fokker-Planck equation (S22) and the transient adjoint equation (S25). We use a Fourier-Galerkin solver for the steady-state equation and a simple finite-difference method for the adjoint problem.

**Steady-state equation** For the forward solution of the stationary Fokker-Planck equation we use a Fourier-Galerkin solver inspired by the Hermite-Galerkin solver introduced by (*59*). Although a Hermite basis is perhaps more appropriate for unbounded state spaces, we found that a Fourier basis solver was sufficient for the present purposes, while avoiding the complication of a non-uniform domain discretization.

We give the detailed derivation in one dimension; extension to higher dimensions is straightforward. The steady-state Fokker-Planck equation for scalar $x$ is

$$0 = -\frac{\partial}{\partial x} f(x)p(x) + \frac{\partial^2}{\partial x^2} a(x)p(x). \tag{S29}$$

With the Fourier representation

$$p(x) = \frac{1}{2\pi} \int_{-\infty}^{\infty} \hat{p}(k) e^{ikx} \mathrm{d}k, \qquad \hat{p}(k) = \int_{-\infty}^{\infty} p(x) e^{-ikx} \mathrm{d}x, \tag{S30}$$

the Galerkin residual is

$$R(k) = \frac{1}{2\pi} \int \hat{p}(k) \mathrm{d}k \left[ -\frac{\partial}{\partial x} \left( f(x) e^{ikx} \right) + \frac{\partial^2}{\partial x^2} \left( a(x) e^{ikx} \right) \right]. \tag{S31}$$

Minimizing the residual requires that it is orthogonal to the subspace spanned by the Fourier modes. Projecting onto an arbitrary wavenumber $k'$ and simplifying with the orthogonality of the Fourier modes,

$$0 = \int \hat{p}(k) \mathrm{d}k \left[ -ik' \hat{f}(k' - k) - k'^2 \hat{a}(k' - k) \right]. \tag{S32}$$

This equation must be true for all $k'$. Practically, the discrete Fast Fourier Transform is used, so the integral is approximated with a sum over wavenumbers.

This gives a linear equation of the form $L\hat{p} = 0$, which is trivially satisfied by $\hat{p} = 0$. However, since the normalization condition implies $\hat{p}(0) = 1$, the corresponding column of $A$ can be moved to the other side of the equation, leaving an inhomogeneous problem:

$$b(k') = \sum_{k \neq 0} L(k', k)\hat{p}(k) \tag{S33}$$

with

$$L(k', k) = -ik'\hat{f}(k' - k) - k'^2\hat{a}(k' - k) \tag{S34a}$$

$$b(k') = ik'\hat{f}(k') + k'^2\hat{a}(k'). \tag{S34b}$$

Finally, we mention an alternative solution method which we found to be more efficient for three-dimensional (or larger) problems. If the Fokker-Planck operator $\mathcal{L}$ is discretized into an array $L$ with any method, the steady-state equation is the homogeneous problem $Lp = 0$ subject to the normalization condition $\int p \, \mathrm{d}x = 1$. An alternative view of this problem is that $p$ is a normalized eigenvector of $L$ with zero eigenvalue. Instead of inverting $L$ as in the method described above, an iterative eigenvalue solver can be used to find the eigenvector corresponding to the smallest real eigenvalue, using for instance an Arnoldi or Krylov-Schur algorithm.

**Adjoint equation** For given drift and diffusion functions $\boldsymbol{f}(\boldsymbol{x})$ and $\boldsymbol{a}(\boldsymbol{x})$, the adjoint Fokker-Planck operator is

$$\mathcal{L}^\dagger = f_j(\boldsymbol{x})\frac{\partial}{\partial x_j} + a_j(\boldsymbol{x})\frac{\partial^2}{\partial x_j^2}. \tag{S35}$$

We discretize both derivatives with second order central finite differencing. The standard derivation of the adjoint problem (*?, 27*) leaves boundary conditions ambiguous, but since the state space is unbounded we apply second-order extrapolation boundary conditions at the edge of the discretized domain.

We solve the transient equation with a matrix exponential of $L^\dagger$, the discretized adjoint Fokker-Planck operator:

$$w_i^{(n)}(\tau) = e^{\tau L^\dagger} x_i^n. \tag{S36}$$

Computing the matrix exponential $e^{\tau L^\dagger}$ destroys the sparse structure of $L^\dagger$ and is relatively computationally expensive, but is fast enough for the one- and two-dimensional problems required for this work. In higher dimensions, a standard time-marching scheme could be applied to leverage the sparse $L^\dagger$.

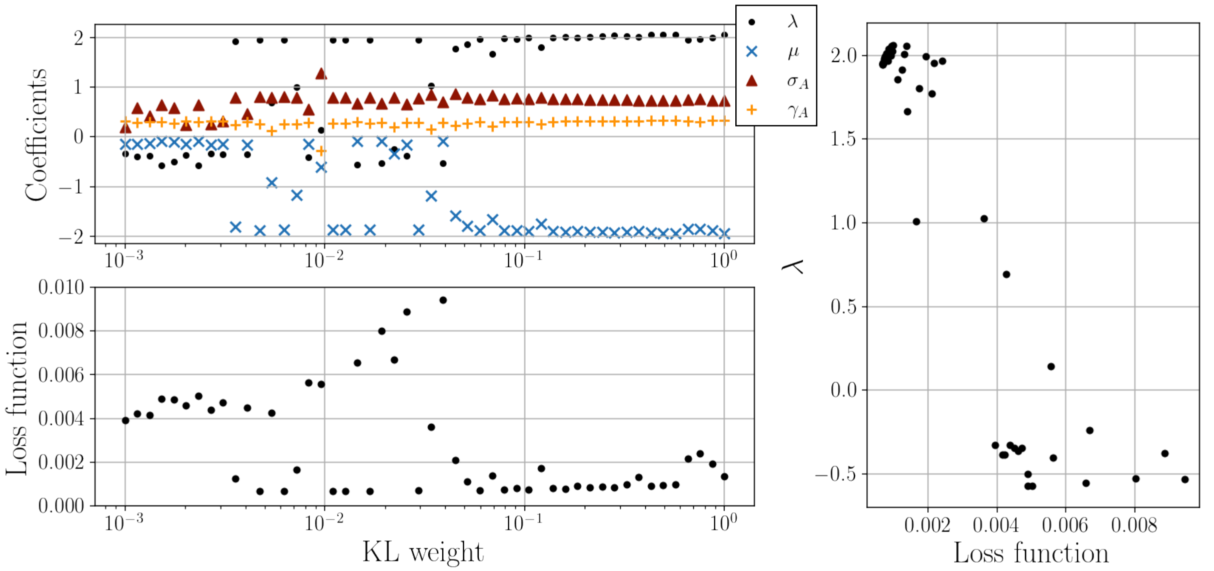


**Supplemental Material, Figure S3:** **Sensitivity with respect to Kullback-Liebler weight.** This weight controls the relative importance of matching the PDF compared to the finite-time conditional moments. The optimization tends towards two clusters of parameter values; for low KL weight most identified models are linearly stable, while for higher weights the models are Stuart-Landau-type equations. As seen by the varying loss function values between these clusters, the higher KL weight helps the optimization avoid the local minimum of the stable models.

# S4 Parameter tuning

The modal analysis is based on conditional averaging, where the only choices are the domain discretization (histogram bins) and spline parameters for interpolating the symmetric deformation. The histogram bins were chosen by balancing spatial resolution with statistical convergence, while the spline parameters were chosen to both fit the conditional average and provide a reasonable estimate of the first derivative for estimating the mean flow modification $\psi_B$. In particular, we chose a quadratic spline with weights given by the inverse of the standard error of the conditional mean in each histogram bin (the same as $W_i^{(n)}$ in Eq. (S28)). Since all these are numerical parameters without physical meaning, we do not observe a strong sensitivity to modest changes.

On the other hand, the Langevin regression method involves choosing two key parameters that can significantly influence the results: the relative weight of the Kullbeck-Leibler (KL) divergence compared to the error in finite time conditional moments, and the coarse sampling rate. Both of these are explored in some depth in (*25*), which introduced Langevin regression.

**Loss function weighting** The the KL weight determines the relative importance of matching the steady-state PDF compared to the short-time statistics. To explore the sensitivity with respect to the KL weight, we repeated the regression for the dynamics of the principal order

parameter $A(t)$ with values of the weight varying over two orders of magnitude, from $10^{-3}$ to $10^{-1}$. Evidently the results vary significantly with different values of the KL weight, although we observe that the coefficient values tend to fall into two clusters. In one, the real parts of both the linear and cubic drift terms are negative, corresponding to a linearly stable model. In the other, the real part of the linear coefficient is positive, indicating that the the symmetric state is linearly unstable.

Physically, the latter situation is the correct one; since the cost function values are consistently lower in this case the unphysical and non-optimal linearly stable solutions are most likely local minima in which the Nelder-Mead simplex optimizer finds itself trapped. Further evidence for this is that increasing the KL weight, adding *more* to the loss function, can lead the optimizer to a lower overall value of the loss compared to excluding the KL divergence term altogether. When the algorithm linearly unstable models are identified, they are largely insensitive to the KL weight. In other words, for small nonzero weights the KL divergence term appears to guide the optimization towards the global minimum of the cost function, without significantly altering its location.

**Coarse sampling rate** The coarse sampling rate is of fundamental importance in system identification methods based on the Kramers-Moyal average (*25, 27*). Formally, the finite-time conditional moments used in the Kramers-Moyal average only converge to the drift and diffusion coefficients of a stochastic process if *(i)* the process is driven by Gaussian white noise, and *(ii)* the sampling rate is infinitely fast. Practically, for developing turbulence models from experimental data we must circumvent both requirements, since even the fast scales in turbulence are not Gaussian white noise, and in any case the sampling rate is fixed experimentally.

Deliberately subsampling the time series tends to "whiten" the apparent forcing by allowing the small, fast scales of the turbulence to decorrelate. Some methods for determining an appropriate coarse sampling rate are given in (***?***, *25*). However, the price for this coarse sampling is distortion in the Kramers-Moyal average, complicating the estimation of drift and diffusion functions (*26*). The precise finite-time distortion can be determined for a given stochastic dynamical system by solving an adjoint Fokker-Planck equation (*?, 27*), which is at the core of the Langevin regression algorithm.

Selecting an appropriate subsampling rate is a matter of balancing decorrelation in the fast time scales with retaining meaningful correlations in the slow, coherent structure dynamics. If the sampling rate is too fast, short-time correlations in the turbulence will destroy the Kramers-Moyal average, while if the sampling frequency is lower than any meaningful frequency relevant for the coherent structures, the Kramers-Moyal average will again tend to zero (*25*). Since turbulence is multiscale in space and time, there is no "spectral gap" by which this determination can be naturally made.

One natural tool for selecting the sampling rate is the autocorrelation function. For a zero-mean variable $X$ governed by a stationary stochastic process the normalized autocorrelation

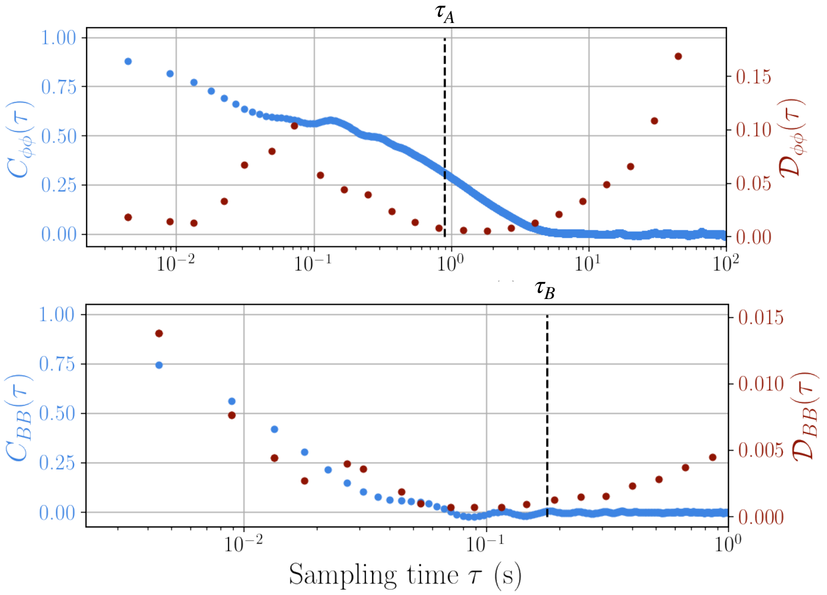


**Supplemental Material, Figure S4: Determiniation of coarse sampling rates.** The $\tau$ values should balance some level of decorrelation ($C(\tau) < 1$) with a sampling rate that approximates a Markov proceess ($\mathcal{D}(\tau)$ near its minimum). The values can be selected independently for $A$ and $B$, but we opt to try and keep the sampling rates relatively nearby for consistency.

function $C_{XX}(\tau)$ is defined as

$$C_{XX}(\tau) = \frac{\langle X(t)X(t+\tau)\rangle_t}{\langle X(t)X(t)\rangle_t}. \tag{S37}$$

Ideally the autocorrelation function will have begun to decay at the selected sampling rate, but will not have completely vanished.

Another helpful diagnostic is the "Einstin-Markov" test described in (*?*). As recognized by Einstein in his work on Brownian motion, no physical process is truly white-in-time when described in continuous time. Therefore there are always unresolved microscopic degrees of freedom, so that any macroscopic model is technically non-Markovian with a fast enough sampling rate (*58*). For classical nonequilibrium statistical mechanics systems like Brownian motion, the characteristic time scale is simply small enough to be irrelevant in practice. The Einstein-Markov test consists of explicitly testing the Markov assumption that the system is memory-less, i.e. that the future state of the system depends only on the current state.

The Markov property implies the following relationship between the three-time PDF and two-time conditional PDFs:

$$p(\boldsymbol{x}'', t+\tau; \boldsymbol{x}', t; \boldsymbol{x}, t-\tau) = p(\boldsymbol{x}'', t+\tau | \boldsymbol{x}', t) p(\boldsymbol{x}', t; \boldsymbol{x}, t-\tau). \tag{S38}$$

This equality can be evaluated by constructing approximations to the left- and right-hand side PDFs with multidimensional histograms and computing the Kullback-Leibler divergence $\mathcal{D}_{KL}(\tau)$

| $\tau_A$ (sec) | $\lambda$ | $\mu$ | $\sigma_A$ | $\gamma_A$ |
|---|---|---|---|---|
| $0.44$ | $3.1$ | $2.9$ | $1.0 + 0.6i$ | $0.2 + 0.5i$ |
| $\mathbf{0.88}$ | $\mathbf{1.9}$ | $\mathbf{1.9}$ | $\mathbf{0.8 + 0.7}\boldsymbol{i}$ | $\mathbf{0.3 + 0.3}\boldsymbol{i}$ |
| $2.22$ | $1.5$ | $1.4$ | $0.6 + 0.6i$ | $0.3 + 0.3i$ |

Table S1: **Langevin regression results for $A$ order parameter with varying coarse subsampling rate.** The selected value $\tau_A = 0.88$ seconds, corresponding to 200 samples at 225 Hz, is shown in bold.

| $\tau_B$ (sec) | $\alpha$ | $\sigma_B$ | $\gamma_B$ |
|---|---|---|---|
| $0.04$ | $66.8$ | $10.3$ | $0.8$ |
| $\mathbf{0.22}$ | $\mathbf{26.4}$ | $\mathbf{6.9}$ | $\mathbf{0.2}$ |
| $0.44$ | $13.5$ | $4.8$ | $0.1$ |

Table S2: **Langevin regression results for $B$ order parameter with varying coarse subsampling rate.** The selected value $\tau_B = 0.22$ seconds, corresponding to 50 samples at 225 Hz, is shown in bold.

between the two. If $\tau$ is large enough that the turbulence is approximately uncorrelated, the KL divergence reaches a minimum before increasing again as the Markov property is again violated by under-resolving the order parameters themselves in time.

Due to the high-dimensional histograms, the computational expense of this test scales very poorly with increasing dimension of $\boldsymbol{x}$ and it is typically only computed for scalar variables. Fig. S4 compares the Einstein-Markov test with the autocorrelation function for the complex phase of $A$ (which has a much longer correlation time than the amplitude) and $B$. Ideally the sampling rate $C(\tau)$ would take on intermediate values and $\mathcal{D}_{KL}(\tau)$ would be near its minimum. However, since the two order parameters $A$ and $B$ evidently must be sampled on different time scales, we sampling rates shown by dashed lines in Fig. S4. In particular, we subsampled at 200 discrete samples at 225 Hz for $A$ and 50 for $B$. Qualitatively, the results are fairly robust to this choice, although the specific coefficient values do change systematically with sampling rate, as shown by the results in Tables S1 and S2.

# S5 Statistical convergence

A key step in data-driven or machine learning-based modeling is confirming that the model is not overfit. In other words, it must not be the case that the model is capable of describing the observed data, but fails on unseen samples drawn from the same ensemble. This is particularly important for highly expressive models such as those used in deep learning. In that high-dimensional context it is also often difficult to quantify the ensemble from which the data is drawn. For example, it is nearly impossible to construct a histogram or empirical PDF over the space of pixelated images without taking recourse to additional models such as variational

autoencoders.

This problem can be largely circumvented with cross-validation, wherein for instance a single data set is split into training and testing subsets; the parameters of the model are optimized against the training data while the performance of the optimized model is evaluated against the withheld test data. Provided the data set is large enough, the working assumption is that both the training and test sets contain representative samples from the unknown underlying distribution.

Although the models we employ in this work are "data-driven", they are of a somewhat different nature. In particular, while the 64-dimensional pressure tap measurements are relatively high-dimensional, it is possible to perform physically-motivated dimensionality reduction based on the behavior of interest (the global instability that breaks axial symmetry, in this case). The parametric modal expansion we introduce, including the conditional averaging and residual projection, captures this process with only three independent degrees of freedom: $\mathrm{Re}(A)$, $\mathrm{Im}(A)$, and $B$.

Following this dimensionality reduction, Langevin regression only has to contend with a low-dimensional state space, which can be characterized relatively easily compared to the space of pixelated images, for instance. SINDy-type sparse polynomial regression further constrains the output of the Langevin regression model. For instance, considering only the drift component of the identified model:

$$\dot{A} = \lambda A - \mu A|A|^2 \tag{S39a}$$

$$\dot{B} = -\alpha B \tag{S39b}$$

with positive $\lambda$, $\mu$, and $\alpha$, it can easily be shown that $B$ is asymptotically stable, while the origin of $A$ is linearly unstable with growth rate $\lambda$ leading to a stable nonlinear equilibrium at $|A| = \sqrt{\lambda/\mu}$. Of course the stochastic model complicates this analysis to some extent, but the probability distribution can still be computed numerically for the entire state space using the Fokker-Planck equation. Since the model can be completely characterized throughout the state space, we claim that the full mean field model must generalize to unseen measurements.

Moreover, Langevin regression is different from most time series forecasting algorithms in that it is not based on predicting either the future state of the system or the time derivative of the present state based on information about the current (and possibly past) states. Rather than using an objective function based on, for instance, mean-squared error evaluated over samples in time, the objective function in Langevin regression directly compares the numerically computed statistics of the proposed model (via the forward and adjoint Fokker-Planck equations) to the empirical statistics. Rather than splitting the data for cross-validation, it is more meaningful to test for statistical convergence.

Figure S5A-B shows the mean and root-mean-square value of measurements collected from one of the pressure taps (nondimensionalized by free-stream pressure) as a function of time series length. Both moments are well-converged; the difference between using 20% of the data and the full time series is less than $10^{-3}$ for both quantities. Since all of the statistics used in both the modal decomposition and stochastic modeling are computed from these pressure

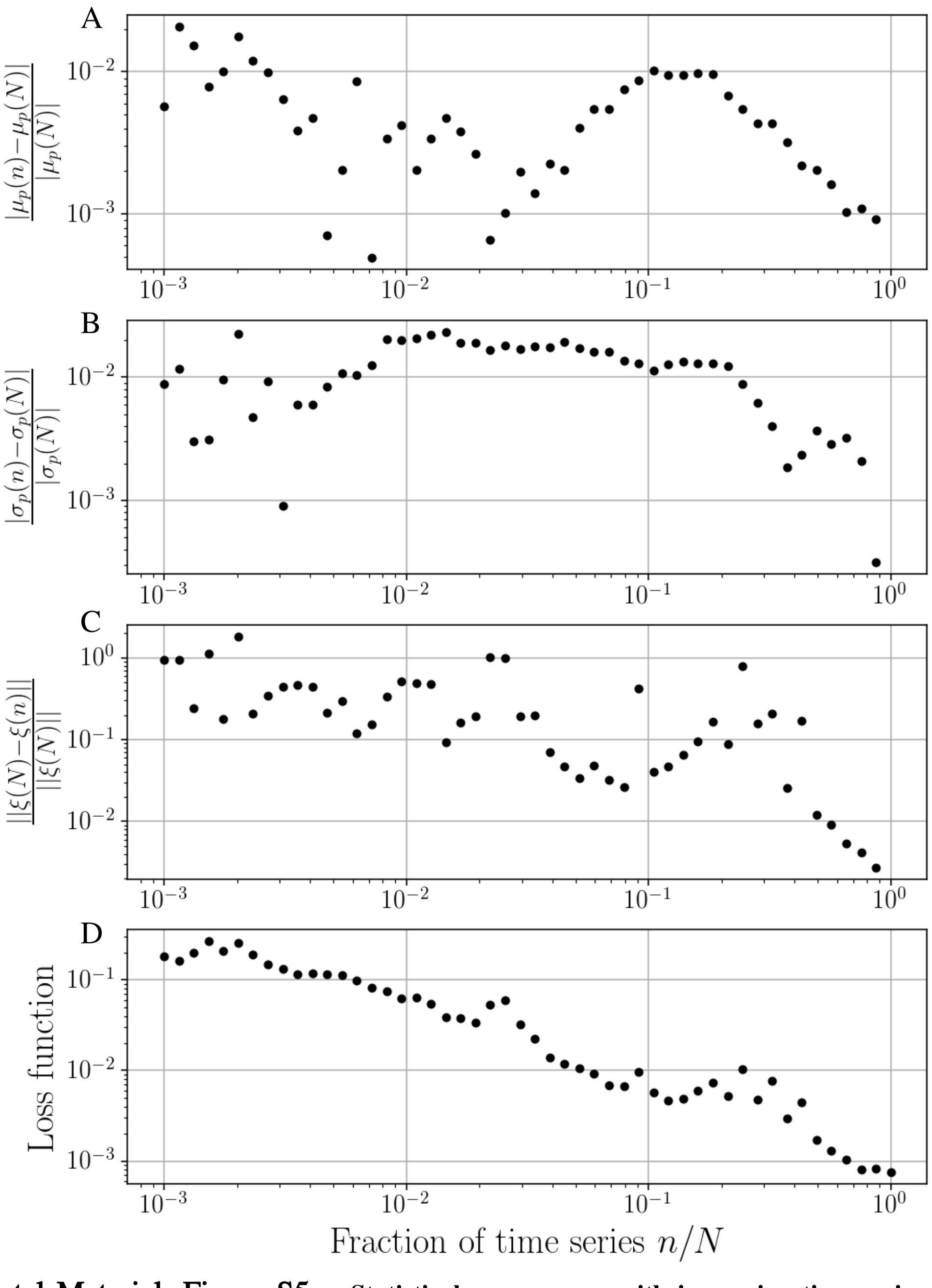


**Supplemental Material, Figure S5:** **Statistical convergence with increasing time series length.** For both the mean (**A**) and root-mean-square deviation (**B**) of an arbitrary pressure sensor, the values using as little as 1% of the data are within $\sim$ 1% of the final value; results are qualitatively similar at all 64 locations. The regression coefficients converge less uniformly, but are also within 1% of their final values with 50% of the data (**C**). Convergence to within 10% is possible with less than 10% of the data, although it is more likely that the optimization will encounter a local minimum, as seen by the increase in both the loss function and relative value of $\xi(n)$ for $n/N$ between 0.01 and 0.5 (**D**).

measurements, this is essential for constructing a model that is consistent with the behavior of the flow. However, since the statistical quantities used in the model are conditional, their convergence could be slower than that of the pressure measurements. To check this, we repeat Langevin regression for the order parameter $A$ using a variable length of the time series. As shown in Fig. S5C-D, the numerical values of the coefficients can be robustly identified with as little as 10% of the data, while both the model coefficients and loss function are essentially unchanged after 80% of the time series. This indicates that all statistical quantities underpinning the stochastic model are well-converged by that point.

The above discussion pertains specifically to models constructed to describe a particular ensemble of flow measurements. Of course, the situation is different if one wishes to design a model that can move *between* ensembles, for instance with varying flow geometry or Reynolds number. In this case, symmetry and scaling considerations suggest that the model would be qualitatively similar for similar flow configurations, but in general both the spatial modes and the Langevin model coefficients would be parametric, e.g. $\psi = \psi(r, A, \mathrm{Re})$ and $\lambda = \lambda(\mathrm{Re})$. Such models can be constructed with data-driven methods like group sparse regression or projection-based modeling; this type of model is of great practical interest but is beyond the scope of the present work.

# S6 Comparison with vector autoregression model

There have been a wide variety of stochastic modeling methods proposed over the years, typically in the context of either system identification or time series forecasting (*32*) . Historically, many of these methods either assumed linear dynamics or were formulated in discrete time. More recently, methods based on Fokker-Planck equations (*?, 24*) and information theory (*30, 60*) have enabled identification of nonlinear Langevin-type stochastic dynamical systems.

In this work we choose to use the Langevin regression framework (*25*), although other methods could also be applied to the stochastic modeling step. For example, this section compares Langevin regression to the widely-used vector autoregression (VAR) approach, which approximates the system with a discrete time model of the form

$$\boldsymbol{x}_k = \boldsymbol{L}_1 \boldsymbol{x}_{k-1} + \boldsymbol{L}_2 \boldsymbol{x}_{k-2} + \cdots + \boldsymbol{L}_p \boldsymbol{x}_{k-p} + \boldsymbol{\Sigma} \boldsymbol{w}_k, \tag{S40}$$

where $\boldsymbol{x}_k$ is the $d$-dimensional state vector at time step $t_k$, each $\boldsymbol{L}_i$ is a separate coefficient matrix, $\boldsymbol{\Sigma}$ is the covariance of the stochastic forcing, and $\boldsymbol{w}_k$ is a $d$-dimensional vector of independent, normally distributed random variables. The model is linear but non-Markovian; the use of the time-delayed "lag" terms up to order $p$ allows the model to potentially capture some of the influence of neglected degrees of freedom. This type of time-delay embedding is often inspired by Takens' theorem in dynamical systems theory or the Mori-Zwanzig formalism in nonequilibrium statistical mechanics (*58*).

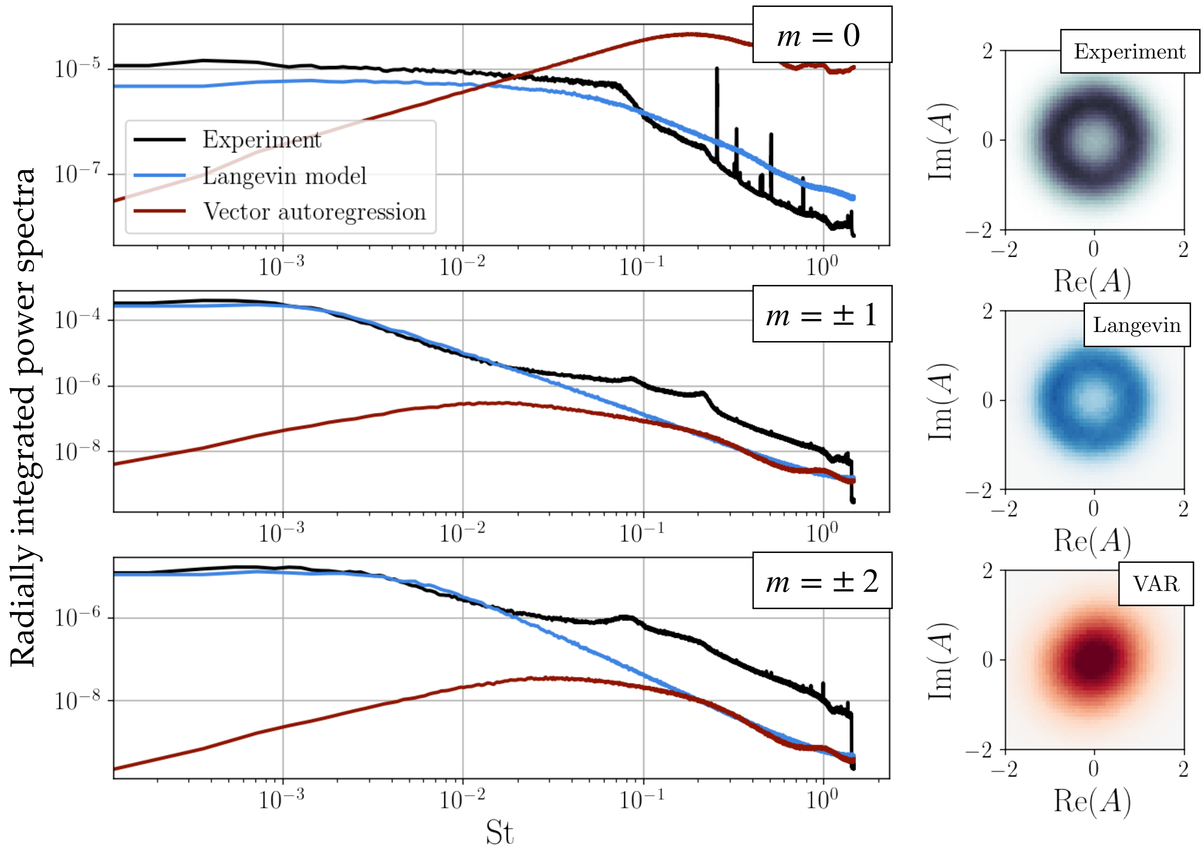


**Supplemental Material, Figure S6**: **Comparison of Langevin regression with vector autoregression (VAR) model.** Although VAR is able to capture non-Markovian behavior by using lag terms, it cannot resolve the nonlinear stability of the symmetry-breaking, as seen in the estimated PDFs (right). The VAR model also predicts the power spectra less accurately than the Langevin regression model.

We fit a three-dimensional VAR model for $\boldsymbol{x} = \begin{bmatrix} \mathrm{Re(A)} & \mathrm{Im(A)} & B \end{bmatrix}^T$ using the Python `statsmodels` package.The only tuning parameter is the order $p$ of the model; we select $p = 5$ based on the Akaike information criteria. Figure S6 compares Monte Carlo simulations of the VAR model and the Langevin regression model to the experimental data. The power spectra for the reconstructed Fourier modes based on the VAR model does not match the experiment as well as the Langevin model, although the Langevin model is also too simple to resolve details in the power spectra at higher frequencies. More importantly, the linear VAR model cannot capture the structure of the probability distribution, which reflects the Stuart-Landau nonlinear stability mechanism that the proposed mean field model was constructed specifically to capture.

This comparison is not meant to suggest that an autoregression-type model cannot be constructed which would describe this behavior. Indeed, the nonlinear autoregressive moving average model with exogenous inputs (NARMAX) framework (*32*) is perhaps the most flexible and general approach to system identification at present. A properly tuned NARMAX model (requiring selection of many more parameters than the lag order $p$ of a VAR model) could well match or exceed the performance of the Langevin model. However, part of the flexibility and generality of NARMAX stems from its formulation in discrete time, while the natural description of the symmetry-breaking behavior in the present case is as a continuous dynamical system. It is not possible in general to convert a discrete time autoregressive model to a standard stochastic differential equation, limiting its interpretability. For these reasons we opt to use Langevin regression and note that the best choice of system identification method will be application-specific.

# S7 Influence of measurement noise

Control theory distinguishes between two types of noise present in engineering systems: process noise and measurement noise. For a system governed by a Langevin equation, these can be separated by introducing a measurement function:

$$\dot{x} = f(x) + \sigma_x(x) w_x(t) \tag{S41a}$$

$$z = g(x) + \sigma_z(x) w_z(t). \tag{S41b}$$

Both process and measurement noise can be problematic for model discovery, especially for algorithms that rely on approximating derivatives from noisy dataMany of these methods also assume deterministic dynamics; treatments of noise therefore focus exclusively on measurement noise.

In contrast, Langevin regression allows for and to some extent relies on process noise. However, in this work we have so far ignored measurement noise, assuming that $g(x) = x$ and $\sigma_z = 0$. A degree of robustness to experimental errors in the pressure taps is built into the analysis with conditional averaging and numerical integration, but it is difficult to analytically propagate measurement noise through this analysis. Furthermore, although the process noise

(turbulence) is known to violate the assumption of Gaussian white noise, it is not clear what the impact of the same departure in measurement noise would be.

Here we briefly explore these questions by adding artificial measurement noise generated from both Gaussian and non-Gaussian distributions and propagating this through the identification of a simple Langevin model for the radial location of the center of pressure $|A|$. Of course, since the original data was from experimental measurements, there is inevitably already some degree of measurement noise. Since this cannot easily be quantified, for the purposes of this section we treat the experimental data as "truth".

We add independent, identically distributed measurement noise to each of the 64 pressure tap measurements, as shown in Fig. S7. The noise is drawn from either a standard normal or standard Cauchy distribution and scaled by a factor $\eta$ of the standard deviation of the pressure measurements across all taps. The Cauchy distribution is a classic example of a "heavy-tailed" distribution with extreme values. We then compute the center of pressure, empirical PDF, and Kramers-Moyal coefficients as usual and assume the form of the dynamics identified in an earlier work (*25*):

$$\frac{\mathrm{d}|A|}{\mathrm{d}t} = \lambda|A| - \mu|A|^3 + \frac{(\sigma + \gamma|A|^2)^2}{2|A|} + (\sigma + \gamma|A|^2)w(t). \tag{S42}$$

Fig. S7A-B shows the relative error in identified Langevin coefficients with added Gaussian and Cauchy noise, respectively. The method is relatively robust to Gaussian noise until a signal-to-noise ratio of $\eta = 1$. In contrast, much lower levels of Cauchy noise, nearly invisible in the time series (Fig. S7C) can disrupt the algorithm.

The statistics, shown in Fig. S7D-F, illuminate the different failure modes for Gaussian and Cauchy noise. For Gaussian noise, the Kramers-Moyal average tends to filter out moderately high levels of noise, but eventually the noise level is high enough that the PDF becomes distorted (Fig. S7D). On the other hand, the Kramers-Moyal average appears to be much more sensitive to the extreme values of the Cauchy noise, particularly for the diffusion (note the log scale in Fig. S7F). This suggests that relatively low levels of strongly non-Gaussian measurement noise can lead to failure, even though the Langevin regression method only formally assumes that the *process* noise is Gaussian. Fortunately, this issue appears easy to diagnose since in this case the estimates of the diffusion $\hat{a}$ are completely inconsistent with fluctuations in the time series. For the purposes of the experimental data used in this work, we conclude that any measurement noise is at least approximately Gaussian.

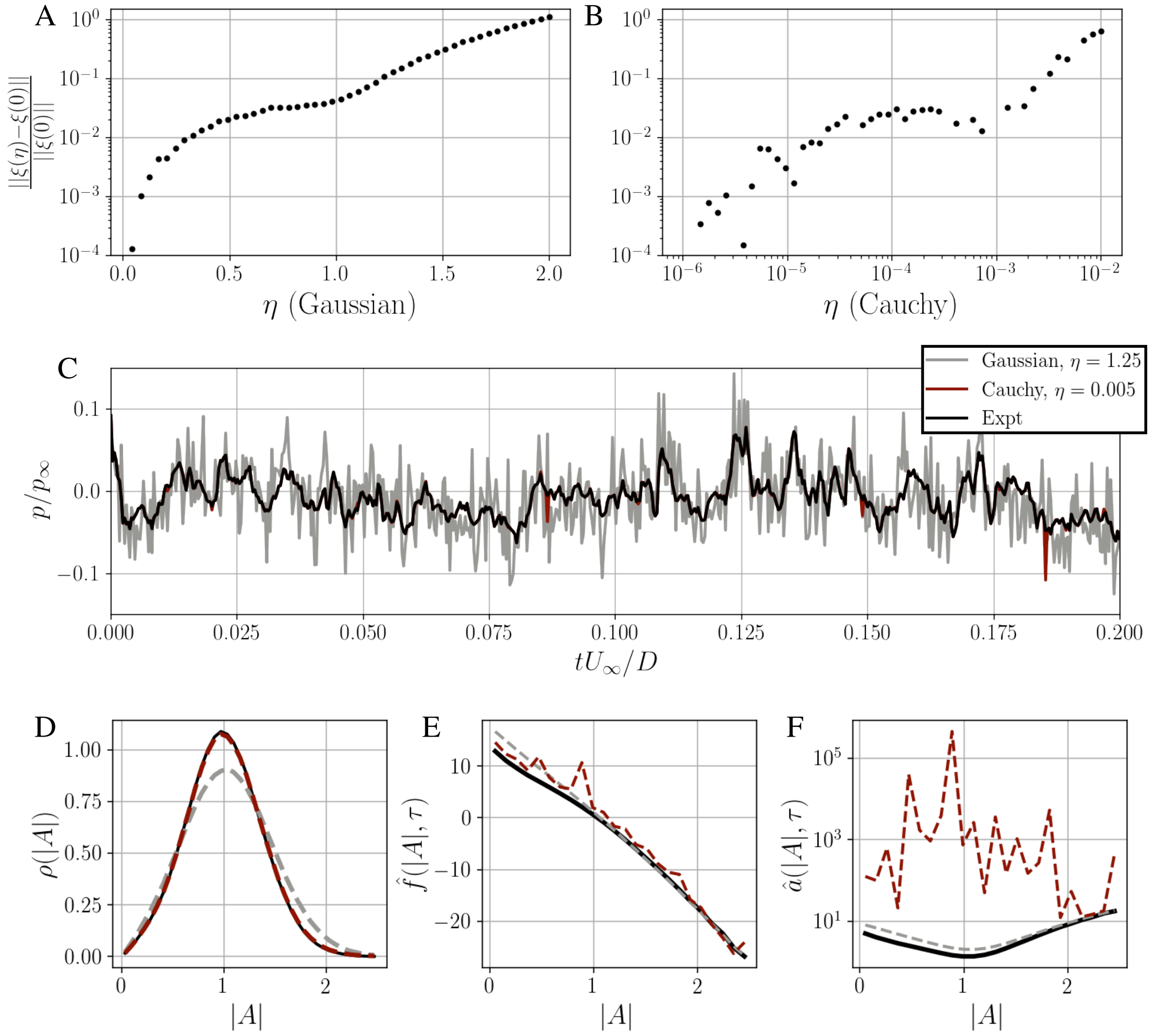


**Supplemental Material, Figure S7:** **Sensitivity with respect to measurement noise.** Randomly generated, independently distributed artificial noise is added to each pressure sensor and propagated through a model for the order parameter amplitude $|A|$. For both Gaussian and Cauchy-distributed noise, the amplitude is $\eta\sigma_p$, where $\sigma_p$ is the root-mean-square value of the measured pressure fluctuations. The results are robust both Gaussian and non-Gaussian noise for modest values of $\eta$ (**A**-**B**), algorithm begins to fail at much lower noise levels for the heavy-tailed Cauchy noise (shown for an example sensor time series in **C**), primarily due to worse estimates of the Kramers-Moyal average $\hat{f}$ and $\hat{a}$ compared to Gaussian measurement noise (**D**-**F**).
